# Cold Exposure-Induced Intestinal Dysfunction: Mechanisms, Animal Models, and Nutritional Intervention Strategies

**DOI:** 10.3390/nu18203350

**Published:** 2026-10-13

**Authors:** Shujie Guo, Zhanhui Geng, Jingman Zhang, Beibei Dou, Yang Liu, Xiaonan Wang, Yingze Jiao, Qianwen Li, Han Chen

**Affiliations:** 1 Food Laboratory of Zhongyuan, Henan University of Technology, Zhengzhou 450001, China; guoshujie@stu.haut.edu.cn (S.G.); jmzhang@stu.haut.edu.cn (J.Z.); 2 Academy of Military Sciences, PLA, Systems Engineering Institute, Beijing 100010, China; zhanhuigeng@163.com; 3 Food Laboratory of Zhongyuan, Luohe 462000, China; doubeibei@zyfoodlab.cn (B.D.); liuyang@zyfoodlab.cn (Y.L.); wangxiaonan@zyfoodlab.cn (X.W.); jiaoyingze@zyfoodlab.cn (Y.J.); liqianwen@zyfoodlab.cn (Q.L.); 4 Food Laboratory of Zhongyuan, China Agricultural University, Beijing 100080, China

**Keywords:** cold exposure, intestinal barrier, gut microbiota, oxidative stress, inflammation, animal models, nutritional interventions, food-derived bioactive compounds

## Abstract

Cold exposure is an important environmental stressor that can disrupt physiological homeostasis and induce complex metabolic, immune, oxidative, and gastrointestinal responses. Increasing evidence indicates that the intestine is a sensitive target of cold exposure, yet the underlying mechanisms and their interrelationships remain incompletely understood. This review summarizes and critically evaluates current evidence on cold exposure-induced intestinal dysfunction and adaptation, with emphasis on intestinal barrier integrity, mucus and digestive function, inflammatory and oxidative responses, pyroptosis-related signaling, gut microbiota remodeling, animal models, and potential intervention strategies. Experimental studies in rodents, pigs, poultry, and fish indicate that cold exposure can induce intestinal morphological alterations, epithelial barrier disruption, increased permeability, tight-junction remodeling, mucus and digestive dysfunction, inflammatory activation, oxidative stress, apoptosis, and, under specific conditions, inflammasome-associated pyroptosis. Concurrent alterations in gut microbial composition and metabolites may interact with epithelial and immune responses, although the temporal and causal relationships among these processes remain insufficiently defined. Importantly, intestinal responses to cold are highly context-dependent and vary according to exposure temperature, duration, pattern, species, nutritional status, acclimation history, and pre-existing disease. Evidence from experimental colitis models further suggests that controlled cold exposure may promote mucosal repair and antimicrobial defense under selected conditions, indicating that cold exposure is not universally detrimental to intestinal health. This review also evaluates nutritional and food-derived strategies, including rutin, plant-derived preparations, dietary glucose, sodium butyrate, vitamin D3, probiotics, prebiotics, and other microbiota-targeted approaches, which have shown potential in mitigating cold-associated intestinal disturbances. Finally, major methodological and translational challenges are discussed, including the need for standardized cold-exposure protocols, causal and longitudinal studies, functional microbiome analysis, and better translation from animal models to human health. Collectively, current evidence supports a multidimensional and context-dependent framework for understanding how cold exposure influences intestinal homeostasis and highlights nutritional and food-derived interventions as promising strategies for maintaining intestinal health under cold environmental conditions.

## 1. Introduction

Cold exposure represents an important environmental challenge that requires coordinated physiological adaptations to maintain thermal homeostasis. When ambient temperature falls below the thermoneutral range, mammals increase heat production and limit heat loss through mechanisms including peripheral vasoconstriction, shivering, and non-shivering thermogenesis [1,2]. Brown adipose tissue (BAT) is a major site of non-shivering thermogenesis, in which sympathetic activation stimulates uncoupling protein 1 (UCP1) dependent mitochondrial heat production [2,3,4]. Cold acclimation can further modify the contribution of shivering and non-shivering thermogenesis and induce metabolic adaptations across multiple tissues [1,2]. However, when the intensity or duration of cold exposure exceeds the capacity of these adaptive responses, substantial alterations in energy metabolism, endocrine regulation, immune function, and tissue homeostasis may occur [1,5]. Importantly, the physiological effects of cold exposure extend beyond classical thermoregulatory organs. Cold-induced sympathetic activation, changes in vascular tone, substrate mobilization, and tissue-specific metabolic remodeling can influence multiple peripheral organs and contribute to systemic physiological responses [2,5]. In this context, the gastrointestinal tract has emerged as an important, but comparatively underappreciated, target of cold-associated physiological stress.

The intestine is particularly sensitive to systemic and environmental disturbances because it integrates nutrient absorption, epithelial barrier maintenance, mucosal immune surveillance, and continuous interactions with the gut microbiota. Intestinal barrier integrity is maintained by a multilayered defense system composed of epithelial cells, mucus-producing goblet cells, antimicrobial factors, and intercellular junctional complexes, including tight-junction proteins such as zonula occludens-1 (ZO-1) and claudins [6,7,8]. The mucus layer provides an additional protective interface between luminal microorganisms and the epithelium, while the gut microbiota contributes to nutrient metabolism, epithelial renewal, immune development, and intestinal homeostasis [7,8,9]. Disruption of these interconnected components can increase epithelial permeability and facilitate host exposure to microbial-associated molecules, thereby promoting mucosal immune activation and inflammatory responses. Consequently, environmental challenges that simultaneously influence host metabolism, immune regulation, oxidative balance, and microbial ecology may have substantial effects on intestinal function.

Accumulating experimental evidence indicates that cold exposure can disturb intestinal homeostasis through multiple interconnected mechanisms. Acute cold-related stress has been associated with intestinal mucosal injury, increased permeability, altered epithelial morphology, and changes in tight-junction-associated proteins in mice [10]. Chronic cold exposure has likewise been shown to impair colonic epithelial integrity, promote inflammatory and endoplasmic-reticulum stress responses, and induce oxidative stress and epithelial apoptosis [11]. In another mouse model, prolonged cold exposure was associated with altered intestinal inflammatory mediators, activation of toll-like receptor 4 (TLR4)/myeloid differentiation factor 88 (MyD88)/nuclear factor-κB (NF-κB) and mitogen-activated protein kinase (MAPK)-related signaling, increased NOD-like receptor thermal protein domain associated protein 3 (NLRP3) inflammasome- and caspase-associated pyroptosis, and substantial changes in gut microbial diversity [12]. Cold-induced intestinal dysfunction has also been reproduced in other species, including poultry and pigs, in which cold exposure has been associated with intestinal structural remodeling, altered barrier-related molecules, inflammatory responses, and metabolic disturbances [13,14,15]. Moreover, a mouse study using repeated intragastric administration of cold water demonstrated increased intestinal permeability, reduced microbial diversity, altered microbial ecological networks, and enrichment of bacteria belonging predominantly to Proteobacteria [16]. Together, these observations suggest that the intestine is not simply a passive downstream target of systemic cold stress but may represent an important site at which metabolic, epithelial, immune, and microbial responses converge.

Importantly, however, the intestinal response to cold exposure does not appear to be uniformly detrimental. The biological consequences of cold depend strongly on exposure intensity, duration, exposure modality, circadian timing, host species and genetic background, nutritional status, acclimation history, and pre-existing disease [1,2,17]. While sustained or severe cold exposure can impair epithelial integrity and enhance inflammatory and oxidative responses in healthy animals, emerging evidence indicates that appropriately controlled cold exposure may exert adaptive or even protective effects under selected pathological conditions. In experimental colitis models, cold exposure has been reported to attenuate intestinal inflammation and preserve epithelial barrier function through mechanisms involving Peroxisome proliferator-activated receptor γ (PPARγ )/NF-κB signaling [18]. More recently, cold exposure was shown to enhance intestinal epithelial proliferation, promote colonic stem-cell-associated regeneration, and strengthen ileal antimicrobial defense in mice with experimental colitis [19]. These findings indicate that cold exposure should not be considered intrinsically harmful or beneficial to the intestine. Rather, its effects appear to depend on the interaction among environmental conditions, host physiological state, exposure history, and intestinal disease status. Defining the determinants of these divergent responses is therefore essential for understanding the biological significance of cold exposure.

Several interconnected pathways may contribute to cold-associated intestinal responses. Increased metabolic demand and oxidative stress during cold exposure may alter epithelial homeostasis and tight-junction organization [11,12]. When epithelial barrier integrity is compromised, increased luminal access of microbial products, including lipopolysaccharide (LPS), may provide stimuli for innate immune pathways such as TLR4/MyD88/NF-κB signaling [12,20]. Activation of inflammatory signaling may in turn interact with MAPK pathways and inflammasome-associated responses, including NLRP3 activation and pyroptosis [12,21]. At the same time, cold exposure can modify gut microbial composition and metabolic activity, including changes in short-chain fatty acids (SCFAs) and other microbial metabolites [22]. These observations support a model of reciprocal interaction in which epithelial dysfunction, microbial disturbance, oxidative stress, and innate immune activation may reinforce one another. Nevertheless, the current evidence does not establish a single linear causal sequence among these processes. In most experimental studies, epithelial, inflammatory, and microbial endpoints are measured at similar time points, making it difficult to determine the temporal hierarchy between dysbiosis, barrier injury, and inflammatory activation. Thus, these pathways should currently be regarded as interconnected and potentially mutually reinforcing responses rather than as a definitively established sequential cascade.

Despite increasing interest in the gastrointestinal consequences of environmental stress, the effects of cold exposure on intestinal health remain incompletely integrated across physiological, epithelial, immune, microbial, and metabolic levels. Considerable heterogeneity exists among acute, chronic, intermittent, and cold–humid exposure models, as well as models combining cold with restraint, immersion, or cold-water administration [10,16,17]. Such heterogeneity complicates direct comparisons and makes it difficult to determine whether intestinal alterations arise primarily from low temperature itself, secondary systemic metabolic adaptation, or interactions with accompanying stressors. Furthermore, the mechanisms underlying the apparently divergent intestinal effects of cold in healthy versus inflammatory conditions remain poorly understood. A more comprehensive framework is therefore needed to integrate cold-induced alterations in intestinal barrier function, mucus-associated defense, digestive activity, innate immunity, oxidative homeostasis, and gut microbial ecology.

**Figure 1:**
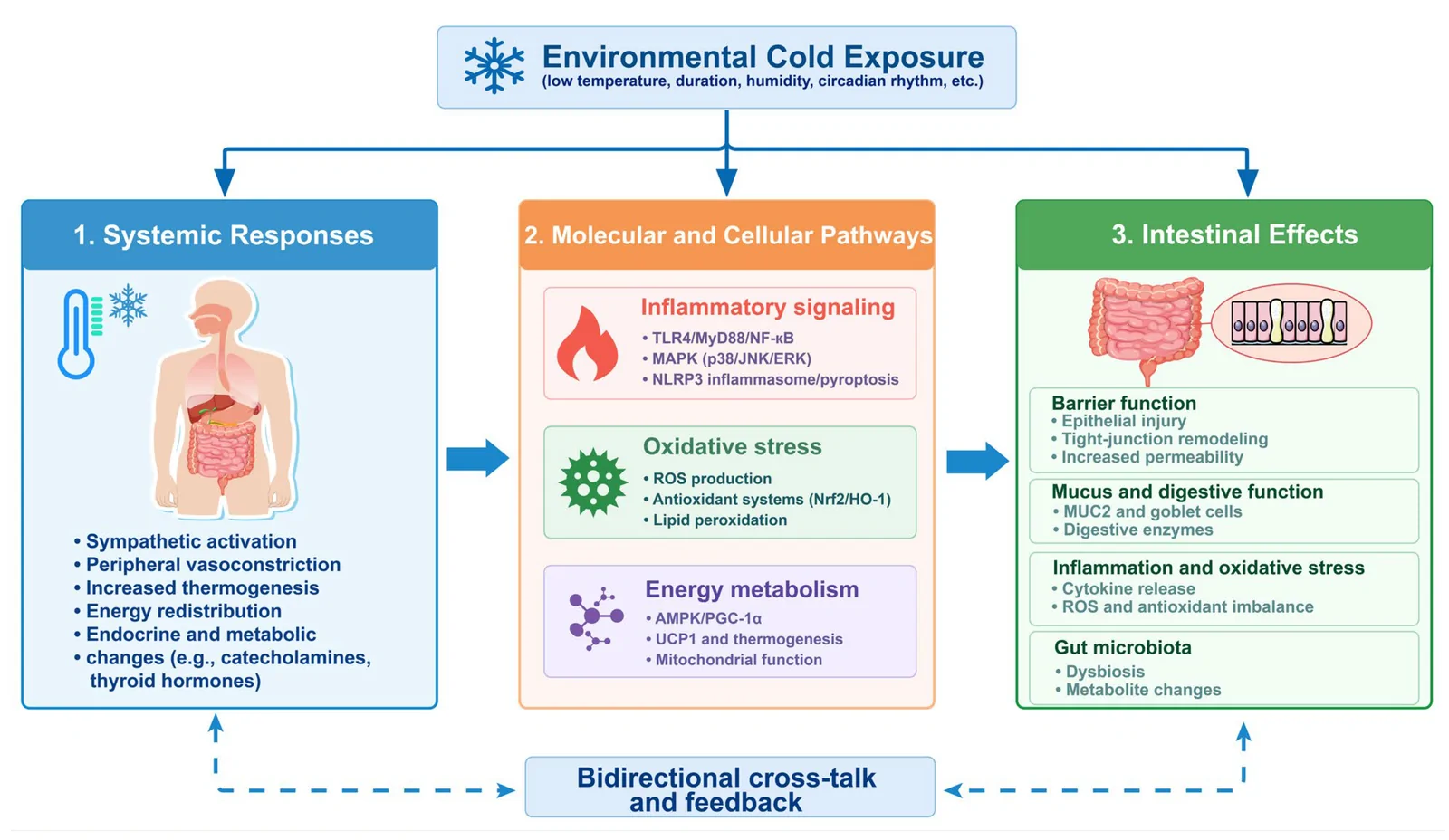
A Multilevel Mechanistic Framework Linking Environmental Cold Exposure to Intestinal Responses.

Accordingly, this review summarizes current evidence regarding cold exposure-induced intestinal dysfunction and adaptation, with particular emphasis on epithelial barrier disruption, mucus and digestive alterations, inflammatory and pyroptotic signaling, gut microbiota remodeling, animal models, and potential nutritional and food-derived intervention strategies. By integrating evidence across mammalian, avian, and aquatic models, this review aims to clarify the major biological pathways linking environmental cold exposure to intestinal homeostasis, distinguish pathological responses from adaptive remodeling, and identify key methodological and mechanistic gaps that should be addressed in future research. Figure 1 illustrates a multilevel framework linking environmental cold exposure to systemic physiological responses, molecular and cellular pathways, and intestinal outcomes, with bidirectional cross-talk and feedback among these processes.

### Literature Search and Selection Strategy

This narrative review was conducted to identify and critically evaluate experimental and translational evidence concerning the effects of environmental cold exposure on intestinal physiology, intestinal barrier function, gut microbiota, and nutritional or food-derived interventions. Literature searches were performed using PubMed/MEDLINE, Web of Science Core Collection, and Google Scholar, with the final search conducted on 15 August 2026.

The search strategy combined terms related to cold exposure and intestinal outcomes, including combinations of “cold exposure,” “cold stress,” “cold acclimation,” “low temperature,” “cold water immersion,” “cold–humid stress,” “intestine,” “intestinal barrier,” “intestinal permeability,” “tight junction,” “MUC2,” “gut microbiota,” “inflammation,” “oxidative stress,” “pyroptosis,” “thermogenesis,” “nutrition,” “food-derived bioactive compounds,” and “nutritional intervention.” Reference lists of relevant articles and recent reviews were also screened to identify additional studies.

Studies were considered relevant when they investigated cold or low-temperature exposure in humans or experimental models and reported outcomes related to intestinal structure or function, epithelial or mucus barriers, digestive function, intestinal immunity, oxidative stress, gut microbiota, or interventions targeting these outcomes. Studies focused exclusively on systemic cold tolerance or non-intestinal outcomes were considered separately and were not used as direct evidence for intestinal protection. Because this article is a narrative review rather than a systematic review or meta-analysis, no formal risk-of-bias scoring or quantitative synthesis was performed. Evidence was interpreted according to the experimental model, exposure protocol, outcome measured, and degree of mechanistic or causal support.

## 2. Mechanisms of Cold Stress-Induced Impairment of Intestinal Barrier Function

Rather than functioning as independent compartments, these components interact continuously to maintain epithelial integrity, regulate intestinal permeability, restrict the access of potentially harmful luminal factors, and coordinate local and systemic immune and metabolic homeostasis [23]. Cold exposure may perturb this integrated system through increased thermogenic and metabolic demands, redistribution of regional blood flow, oxidative stress, neuroendocrine responses, and immune modulation [1,2]. Among these systemic responses, cold-induced hemodynamic redistribution may represent an important upstream mechanism linking whole-body thermoregulation to intestinal dysfunction.

Cold-induced hemodynamic redistribution may directly influence intestinal perfusion. Exposure to cold activates sympathetic–adrenergic pathways and produces vasoconstriction in peripheral vascular beds as part of the thermoregulatory response. Importantly, this response is not restricted to the skin and limbs. In healthy humans, whole-body skin-surface cooling has been shown to reduce vascular conductance in the celiac and superior mesenteric arteries, indicating that splanchnic vasoconstriction accompanies peripheral vasoconstriction during cold exposure [24]. Experimental studies in newborn piglets similarly demonstrated that environmental cold stress increased intestinal vascular resistance and reduced small-intestinal blood flow and oxygen delivery, while cardiac output was redistributed toward thermogenic tissues [25]. These findings indicate that intestinal perfusion may be compromised even during a successful thermoregulatory response, although the magnitude of this redistribution is dependent on the severity and physiological context of cold exposure.

Reduced intestinal perfusion may become particularly relevant when oxygen demand is simultaneously elevated. Cold exposure increases whole-body metabolic requirements and thermogenic activity, potentially increasing the mismatch between intestinal oxygen supply and tissue metabolic demand when splanchnic blood flow is reduced. In piglets, environmental cold stress decreased gastrointestinal blood flow and oxygen delivery, and the reduction in oxygen availability was further exacerbated under anemic conditions [26]. When intestinal oxygen delivery becomes insufficient, mucosal hypoxia and microcirculatory dysfunction may impair epithelial energy metabolism and barrier integrity, increase oxidative stress, and facilitate inflammatory responses [27,28]. Thus, hemodynamic redistribution and tissue oxygenation should be considered as an upstream physiological component of cold-associated intestinal injury, potentially interacting with downstream pathways such as TLR4/MyD88/NF-κB, MAPK, and NLRP3-associated inflammatory signaling rather than acting as an independent mechanism.

**Figure 2:**
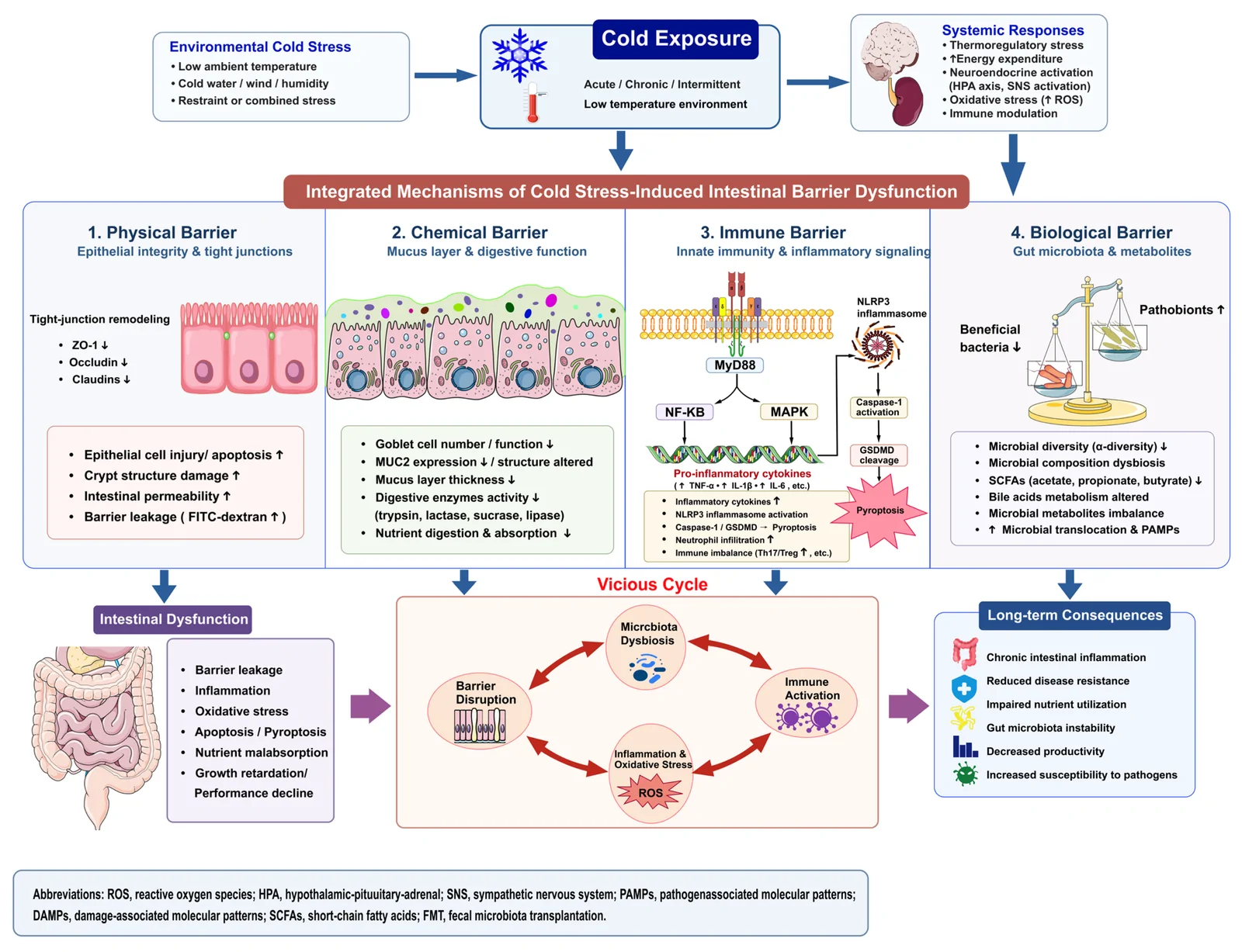
Integrated mechanisms of cold stress-induced intestinal barrier dysfunction and intestinal–microbiota interactions.

Experimental studies have consequently reported cold-associated changes in epithelial morphology and permeability, tight-junction organization, mucus and digestive function, inflammatory signaling, and gut microbial composition, although the magnitude and direction of these responses vary substantially according to exposure conditions and host characteristics [10,11,12,16]. Importantly, these alterations may interact with one another rather than occur independently. Epithelial and mucus disruption may increase host exposure to microbial products, whereas inflammatory and oxidative responses may further compromise epithelial homeostasis; meanwhile, changes in the gut microbiota and its metabolites may influence epithelial metabolism and immune regulation [16]. The current evidence therefore supports an integrated framework in which cold exposure can affect multiple interconnected components of intestinal homeostasis, while the precise temporal and causal relationships among these processes remain to be established. Figure 2 summarizes the proposed mechanisms linking systemic responses to cold exposure with alterations in the physical, chemical, immune, and microbial components of the intestinal barrier.

### 2.1. Physical Barrier: Epithelial Injury, Altered Permeability, and Tight-Junction Remodeling

The intestinal physical barrier is primarily constituted by a continuous layer of intestinal epithelial cells and specialized intercellular junctional complexes that regulate the movement of molecules across the epithelial surface [6,8]. Tight junctions, including ZO-1 and claudins, are essential structural and functional components of this barrier and contribute to the selective regulation of paracellular permeability [29]. The integrity of the epithelial barrier is maintained through continuous epithelial proliferation, differentiation, migration, and shedding along the crypt–villus axis [30]. Accordingly, disruption of epithelial architecture or junctional organization can increase intestinal permeability and expose the mucosa to luminal antigens, microbial products, and other potentially harmful substances [6,8,30].

Accumulating experimental evidence indicates that cold exposure can impair intestinal epithelial integrity and morphology. In a mouse model of acute cold water-immersion restraint (CWIR) stress, cold exposure induced marked intestinal mucosal injury and increased intestinal permeability, accompanied by elevated intestinal tumor necrosis factor-α (TNF-α) expression and reduced gut microbial diversity. Histological analyses demonstrated pronounced alterations in the intestinal mucosa, indicating that acute cold-related stress can rapidly compromise epithelial integrity. Importantly, the same study showed that disruption of the gut microbiota was associated with the severity of intestinal injury, suggesting that epithelial damage and microbial dysbiosis may occur as interconnected responses to acute cold stress [10].

Evidence from genetic intervention studies further supports a functional role of inflammatory signaling in cold-induced intestinal epithelial injury. In mice subjected to acute CWIR stress, genetic deletion of interleukin-6 (IL-6) significantly attenuated intestinal epithelial damage, partially preserved goblet-cell populations and intestinal barrier function, and reduced epithelial apoptosis. These findings provide genetic evidence that IL-6 contributes to the development of intestinal epithelial injury under acute CWIR stress rather than serving solely as an inflammatory biomarker. Notably, the protective effect of IL-6 deficiency against CWIR-induced epithelial apoptosis was associated predominantly with mitochondrial apoptosis-inducing factor (AIF) signaling rather than the canonical caspase-dependent pathway [31]. Collectively, these findings indicate that inflammatory signaling involving IL-6 can participate in the disruption of intestinal epithelial homeostasis during acute cold-related stress, although the contribution of IL-6 may depend on the specific type and severity of stress exposure.

Chronic cold exposure may produce more sustained alterations in epithelial structure and tight-junction integrity. In mice exposed to 4 °C for 3 h/day for 10 consecutive days, chronic cold exposure caused morphological and structural damage in the colon and disrupted the tight junctions of colonic epithelial cells. These changes were accompanied by increased inflammatory responses, activation of the unfolded protein response, epithelial apoptosis, and oxidative stress. Notably, cold exposure inhibited silent information regulator 1 (SIRT1) and promoted p65 acetylation, thereby enhancing inflammatory signaling, while simultaneously affecting nuclear factor erythroid 2-related factor 2 (Nrf2)-associated antioxidant responses. These findings suggest that cold-induced epithelial barrier dysfunction may arise from the interaction of inflammatory, oxidative, and endoplasmic reticulum stress rather than from a single isolated pathway [11].

Alterations in tight-junction proteins have also been documented in avian models, although the direction and magnitude of these changes are not necessarily uniform across experimental conditions. In broilers exposed to 16 ± 1 °C for 72 h, cold stress increased jejunal ZO-1 gene expression and was accompanied by elevated circulating endotoxin concentrations. The authors proposed that these changes in tight-junction-related gene expression reflected remodeling of the intestinal barrier rather than preservation of normal barrier function. This observation is important because it demonstrates that increased transcription of individual tight-junction components does not necessarily indicate improved barrier integrity. Functional measurements of intestinal permeability and bacterial or endotoxin translocation should therefore be interpreted together with molecular measurements of tight-junction proteins [13].

Acute cold stress can also activate stress-responsive pathways upstream of epithelial and junctional injury. In pre-starter broilers exposed to 12 ± 1 °C for 4–12 h, prolonged cold exposure reduced villus height and the villus-to-crypt ratio and increased the expression of cold-inducible RNA-binding protein (CIRP), TLR4, immunoglobulin-regulated enhancer 1 (IRE1), glucose-regulated protein 78 (GRP78), and NF-κB in the ileal mucosa. These findings suggest that cold stress may couple cold-shock responses and innate immune signaling with endoplasmic reticulum stress, thereby contributing to structural intestinal injury [32].

The potential reversibility of cold-induced epithelial dysfunction is supported by recent nutritional intervention studies. In mice exposed to 4 °C for 3 h/day for 21 days, dietary rutin significantly improved the ileal villus-to-crypt ratio and attenuated cold-induced morphological damage. Rutin also improved antioxidant capacity, restored the expression of claudin-1 and ZO-1, reduced inflammatory responses, and inhibited TLR4/NF-κB signaling. These findings support the concept that epithelial barrier dysfunction under cold stress is biologically modifiable and may be targeted through simultaneous regulation of oxidative, inflammatory, and microbial pathways [33].

Overall, current evidence supports a model in which cold exposure disrupts the intestinal physical barrier through multiple interacting processes, including epithelial structural injury, altered permeability, inflammatory activation, oxidative and endoplasmic reticulum stress, and tight-junction remodeling. Importantly, cold-induced changes in ZO-1 and claudins should not be interpreted as uniform or necessarily unidirectional responses. Differences in exposure temperature, duration, stress modality, intestinal segment, species, nutritional status, and pre-existing inflammatory conditions may determine whether tight-junction alterations reflect pathological disruption, compensatory remodeling, or subsequent repair. Future studies should therefore integrate histological analysis with direct measurements of intestinal permeability, tight-junction localization, epithelial apoptosis, and microbial or endotoxin translocation to distinguish adaptive remodeling from functional barrier failure.

### 2.2. Chemical Barrier: Alteration of the Mucus Layer and Digestive Function

The intestinal chemical barrier is formed by mucus, mucins, antimicrobial molecules, and luminal digestive components that collectively protect the intestinal epithelium while supporting nutrient digestion and absorption [7,34]. Among these components, the mucus layer is predominantly composed of the gel-forming mucin Mucin 2 (MUC2) and is continuously secreted by intestinal goblet cells. Rather than functioning as a simple physical coating, the mucus layer forms a dynamic biochemical interface between the host epithelium and the gut microbiota. In particular, the inner mucus layer restricts direct bacterial contact with epithelial cells, whereas the outer mucus layer provides a habitat and nutrient interface for commensal microorganisms [34]. Therefore, disruption of mucus production, secretion, organization, or renewal may increase microbial–epithelial contact and contribute to intestinal inflammation and barrier dysfunction.

Experimental evidence indicates that cold-related environmental stress can alter the intestinal mucus barrier. In mice exposed to cold and humid environmental stress, the mucus layer was significantly reduced, accompanied by decreases in Muc2 content and goblet-cell numbers. Histological analyses further revealed disruption of the mucosal architecture and thinning or loss of the mucus layer. Importantly, experimentally induced microbiota disturbance aggravated the reduction in goblet-cell numbers, mucus-layer thickness, and intestinal Muc2 expression under cold–humid stress. These findings suggest that cold-related mucus barrier injury is not solely an epithelial phenomenon but may involve interactions between the host mucus system and the intestinal microbiota [35].

The effects of cold stress on goblet cells, however, may depend on the intensity and pattern of exposure. In a mouse model of acute CWIR stress, goblet-cell numbers were significantly reduced throughout the duodenum, jejunum, and ileum, together with intestinal mucosal injury and increased permeability [10]. In contrast, a separate study using repeated severe cold exposure (−20 °C for 20 min/day for 2 weeks) reported an increased volume fraction of colonic goblet cells and enhanced mucin production in hypoxia-tolerant mice, suggesting that increased mucus production may represent an adaptive response to repeated cold exposure [36]. These apparently divergent findings indicate that mucus responses to cold exposure are context dependent rather than uniformly suppressive. Differences in exposure temperature, duration, stress modality, intestinal segment, host phenotype, and acclimation status may determine whether alterations in goblet-cell activity and mucin production reflect pathological disruption or adaptive remodeling. Accordingly, changes in goblet-cell number or MUC2 expression should be interpreted together with structural and functional measures of the mucus barrier, including mucus thickness, mucin secretion, epithelial permeability, and microbial localization, rather than being considered isolated indicators of barrier integrity.

The mucus barrier is closely linked to intestinal microbial homeostasis. MUC2-containing mucus contributes to the spatial organization of the intestinal microbiota, limiting microbial penetration toward the epithelial surface while facilitating controlled host–microbe interactions. Disruption of mucus production, organization, or turnover can therefore alter the intestinal microbial environment and increase microbial contact with epithelial and immune compartments [7,34]. In the context of cold and humid environmental stress, multi-omics analyses have suggested that alterations in intestinal mucosal microbiota, particularly changes involving *Lactobacillus reuteri* and host amino-acid metabolism, are associated with intestinal Muc2 expression and gastrointestinal dysfunction. Notably, an integrative model incorporating L. reuteri abundance and host amino-acid metabolites predicted intestinal Muc2 levels with an adjusted R^2^ of 75.0%, suggesting a potential link between microbial composition, host metabolic status, and mucus barrier integrity [35]. However, this regression-based association should not be interpreted as evidence of a direct biochemical or causal relationship between *L. reuteri* and Muc2 regulation. The observed association may reflect coordinated responses to cold–humid stress or other interactions between the microbiota, host metabolism, and intestinal mucosal environment. Therefore, further causal studies using defined microbial communities, microbial depletion or colonization approaches, targeted metabolite supplementation, or gain- and loss-of-function experiments are required to determine whether *L. reuteri* directly influences Muc2 expression and mucus-barrier function.

Cold exposure can additionally influence digestive function through alterations in gastrointestinal motility and secretion. Human experimental data obtained from healthy volunteers demonstrated that a cold-pain stimulus significantly delayed gastric emptying and induced a biphasic change in pancreatic trypsin output, characterized by an initial reduction during cold stimulation followed by increased secretion during the post-stress period. Although this model does not reproduce sustained environmental cold exposure, it provides direct evidence that an acute cold-related stressor can rapidly alter upper gastrointestinal motility and secretory function. The concurrent autonomic responses observed in the same study suggest that these gastrointestinal changes may form part of the broader physiological stress response [37].

Evidence from animal models further indicates that digestive enzyme responses to cold exposure are highly context dependent. In mice exposed to cold and humid environmental stress for 7 days, intestinal sucrase and lactase activities were significantly increased, together with increased microbial activity and marked changes in intestinal microbial community composition [38]. These changes may represent a compensatory alteration in carbohydrate digestive activity under cold–humid stress, although their functional significance remains uncertain. Importantly, increased activity of individual digestive enzymes should not automatically be interpreted as improved overall intestinal function because digestion and nutrient absorption depend on multiple coordinated processes, including intestinal epithelial integrity, substrate availability, gastrointestinal transit, and nutrient transport [39]. In addition, both host-derived and microbial enzymes may contribute to luminal digestive activity, further complicating the interpretation of isolated enzyme measurements [38,39].

Protein digestion may respond differently to chronic cold exposure. In chronically cold-exposed Yorkshire pigs, trypsin activity in the jejunal mucosa showed a tendency to decrease, whereas no significant change was observed in the duodenal mucosa. In contrast, cold-adapted Min pigs showed no significant alterations in trypsin activity in either the duodenal or jejunal mucosa. These findings suggest that the effects of cold exposure on digestive enzyme activity may depend not only on exposure intensity and duration but also on breed-specific capacity for cold adaptation [40]. Consistent with this concept, low-ambient-temperature exposure at 4 °C for 96 h increased trypsin activity in duodenal chyme in certain pig breeds, although the magnitude of the response differed among breeds [41]. Collectively, these observations indicate that digestive responses to cold exposure are not necessarily unidirectional.

The intestine may therefore dynamically adjust its digestive and absorptive functions according to the energetic and physiological demands imposed by cold exposure. Changes in disaccharidase or microbial enzyme activity may represent adaptive or compensatory alterations in digestive capacity under increased metabolic demand, although their contribution to whole-body energy availability remains to be established. Conversely, persistent epithelial injury, impaired digestive secretion, or altered intestinal transit may compromise nutrient digestion and absorption. Importantly, the mucus barrier and digestive system should not be considered independent components of intestinal function. Mucus provides a protective interface between the epithelium and the gut microbiota, while microbiota-derived metabolites can regulate epithelial barrier function, goblet-cell activity, mucin production, and mucosal immune homeostasis [7,42,43]. Cold exposure can also modify microbial composition and metabolite production, providing a potential link between environmental temperature, microbial metabolism, and host energy homeostasis [17]. Thus, cold stress may influence the chemical barrier through an integrated network involving goblet-cell activity, mucin production, digestive enzymes, microbial metabolism, and host energy demands.

Overall, current evidence suggests that cold exposure produces context-dependent remodeling of the intestinal chemical barrier rather than a uniform suppression of mucus and digestive functions. Acute severe cold stress may reduce goblet-cell numbers and compromise mucus integrity, whereas prolonged or repeated exposure may induce compensatory mucus production or adaptive changes in digestive activity. The direction and magnitude of these responses are influenced by exposure characteristics, intestinal segment, species, genetic or breed background, nutritional status, and cold-acclimation capacity. Future studies should therefore combine quantitative assessment of mucus thickness and MUC2 with goblet-cell phenotype, mucin secretion, digestive enzyme activity, nutrient absorption, intestinal transit, and microbiota-derived metabolites to determine whether a given biochemical response represents pathological dysfunction or physiological adaptation.

### 2.3. Immune Barrier: Activation of Innate Immunity, Inflammatory Signaling, and Pyroptosis

The intestinal immune barrier comprises epithelial immune functions, tissue-resident and recruited immune cells, secretory immunoglobulins, pattern-recognition systems, and a network of cytokines that collectively distinguish potentially harmful luminal stimuli from physiological host–microbiota interaction [23,44]. Because the intestinal mucosa is continuously exposed to microbial products and dietary antigens, immune activation is tightly coupled to epithelial and microbial homeostasis. Under physiological conditions, innate immune signaling is tightly regulated, whereas excessive activation can disrupt epithelial integrity, increase inflammatory mediator production, and promote tissue injury [44]. Thus, the intestinal immune barrier should not be viewed as an isolated defense layer but as a dynamic regulatory interface linking microbial sensing, epithelial integrity, inflammation, and tissue repair.

Increasing evidence indicates that cold-related stress can activate intestinal immune responses. In a mouse model induced by intragastric administration of cold water for 14 consecutive days, intestinal permeability was increased, while transcriptomic analysis revealed broad activation of immune-related processes in the colon. Differentially expressed genes were enriched in pathways related to innate and adaptive immune responses, including cytokine–cytokine receptor interactions, leukocyte migration, B-cell activation, complement activation, the intestinal immune network for Immunoglobulin A (IgA) production, and T helper cell 1 (Th1)/T helper cell 2 (Th2)/T helper cell 17 (Th17) cell differentiation. These changes were accompanied by reduced bacterial diversity, alterations in the microbial ecological network, and increased abundance of potentially pathogenic bacteria, particularly members of Proteobacteria [16]. Together, these findings suggest that cold-related stress can induce intestinal immune activation in parallel with alterations in the gut microbial ecosystem.

More direct evidence has been obtained from a chronic environmental cold-exposure model in mice. Mice exposed to 4 °C for 3 h/day for 3 weeks exhibited increased concentrations of multiple inflammatory mediators in the ileum, including TNF-α, interleukin-1β (IL-1β), IL-6, interleukin-18 (IL-18), inducible nitric oxide synthase, cyclooxygenase-2 (COX-2), and high-mobility group box 1. Chronic cold exposure was also associated with activation of TLR4/MyD88-mediated NF-κB and MAPK signaling and enhanced activation of pyroptosis-associated pathways [12]. These findings indicate that persistent cold exposure can induce a coordinated intestinal inflammatory response involving both transcriptional inflammatory signaling and inflammatory cell death rather than merely causing a transient increase in individual cytokines.

The TLR4/MyD88/NF-κB axis has been implicated in intestinal inflammatory responses under cold stress. TLR4 is a pattern-recognition receptor that responds to microbial products and endogenous danger-associated signals and can recruit the adaptor MyD88, leading to activation of downstream interleukin-1 receptor-associated kinase (IRAK)/TNF receptor-associated factors (TRAF) signaling, the inhibitor of kappa B kinase (IKK) complex, inhibitor of NF-κB (IκB) degradation, and NF-κB nuclear translocation. This signaling cascade promotes transcription of inflammatory mediators such as TNF-α, IL-1β, IL-6, COX-2, and inducible nitric oxide synthase (iNOS) [20]. In intestinal tissues exposed to cold stress, increased expression or activation of TLR4, MyD88, NF-κB-related molecules, and downstream inflammatory mediators has been reported in both mouse and broiler models [12,45]. Importantly, current evidence does not demonstrate that low temperature directly activates TLR4 in the same manner as a canonical microbial ligand. Instead, epithelial injury, oxidative stress, altered microbial composition, and the release of endogenous danger signals associated with cold exposure may provide secondary stimuli that engage TLR4-dependent inflammatory signaling.

MAPK signaling may also operate in parallel with innate immune activation during cold-induced intestinal inflammation. The major extracellular regulated protein kinases (ERK), c-Jun N-terminal kinase (JNK), and p38 MAPK branches integrate signals generated by environmental stress, cytokines, reactive oxygen species, and cellular injury [46]. In chronically cold-exposed mice, activation of TLR4/MyD88 signaling was accompanied by increased MAPK activity and inflammatory mediator production [12]. Similarly, acute cold stress in broilers increased intestinal expression of TLR4, MyD88, NF-κB-related molecules, inflammatory cytokines, COX-2, and iNOS [45]. These observations support a model in which cold-associated cellular and microbial disturbances converge on innate immune pathways, with NF-κB and MAPK acting as important transcriptional amplifiers of the intestinal inflammatory response.

Cold-induced immune dysfunction may also involve changes in mucosal immune balance rather than simply excessive production of inflammatory cytokines. In broilers subjected to acute cold stress, intestinal expression of several Th1- and Th17-associated cytokines, including IL-1β, interleukin-2 (IL-2), interleukin-8 (IL-8), interleukin-12 (IL-12), interferon-gamma (IFN-γ), and IL-17, was increased, whereas changes were also observed in Th2-associated cytokines and intestinal secretory IgA. These findings suggest that severe cold exposure can disturb local mucosal immune homeostasis. Notably, intermittent mild cold acclimation before an acute cold challenge attenuated several inflammatory changes, reduced activation of the TLR4/MyD88/NF-κB pathway, and improved immune and antioxidant parameters [45]. This observation suggests that previous exposure history may modify the threshold and magnitude of the intestinal inflammatory response to subsequent cold stress.

In addition to transcriptional inflammatory signaling, increasing evidence implicates inflammasome activation and pyroptosis in cold-associated intestinal injury. The NLRP3 inflammasome is a cytosolic innate immune platform consisting primarily of NLRP3, the adaptor apoptosis-associated speck-like protein containing a CARD (ASC), and caspase-1. Following appropriate priming and activation signals, NLRP3 promotes caspase-1 activation, maturation of IL-1β and IL-18, and cleavage of gasdermin D (GSDMD), which forms membrane pores and mediates pyroptotic cell death. In the intestine, inflammasome activation can be triggered by microbial products, mitochondrial dysfunction, reactive oxygen species, ion imbalance, and other cellular danger signals [21,47]. Thus, oxidative stress, epithelial damage, and microbial dysbiosis associated with cold exposure may provide convergent signals capable of promoting inflammasome activation.

Direct evidence for this mechanism has been provided by chronic cold-stress studies in mice. Persistent exposure to 4 °C for 3 h/day for 3 weeks increased inflammatory mediators and promoted GSDMD cleavage through activation of the NLRP3/ASC/caspase-1 axis as well as the caspase-11 pathway. The same study identified concurrent activation of TLR4/MyD88/NF-κB and MAPK signaling, suggesting that transcriptional inflammatory priming and inflammatory cell death may coexist during chronic cold-induced intestinal injury [12]. These observations are consistent with a model in which epithelial or microbial-derived danger signals activate innate immune pathways, while subsequent inflammasome activation promotes IL-1β/IL-18 maturation and GSDMD-dependent pyroptosis. However, the available evidence does not yet establish a universal linear sequence linking TLR4 activation, MAPK signaling, inflammasome assembly, and epithelial injury in all cold-exposure models.

The relationship between intestinal inflammation and epithelial injury is likely bidirectional. Increased inflammatory cytokines and inflammatory cell death may impair epithelial viability and barrier function, whereas barrier disruption can facilitate penetration of microbial products and endogenous danger signals into the mucosal compartment, thereby further stimulating pattern-recognition receptors and inflammasomes [21,23]. Cold exposure may consequently establish a self-reinforcing interaction among epithelial injury, microbial disturbance, innate immune activation, and inflammatory cell death. Nevertheless, most available studies have evaluated these processes at a limited number of time points, and the causal ordering of barrier disruption, microbial dysbiosis, TLR4 activation, and inflammasome assembly remains incompletely defined.

It is also important to distinguish pathological inflammation from physiological cold adaptation. Not all cold exposure produces persistent immune activation. Intermittent mild cold stimulation before acute cold challenge can attenuate intestinal TLR4/MyD88/NF-κB activation and inflammatory cytokine responses in broilers while improving antioxidant and immune parameters [45]. This suggests that the intestinal immune response depends on the intensity, duration, frequency, and history of cold exposure. Severe or prolonged exposure may exceed adaptive capacity and promote pathological inflammation, whereas appropriately controlled repeated exposure may induce a more tolerant phenotype. However, whether these effects represent a generalized hormetic response remains uncertain and requires standardized dose–response and longitudinal studies.

Overall, current evidence supports a multi-stage model of cold stress-associated intestinal immune dysfunction. Cold exposure can promote epithelial injury, oxidative stress, and microbial perturbation, which may generate endogenous and microbial danger signals. These signals can engage TLR4/MyD88-dependent NF-κB and MAPK signaling, increase production of inflammatory mediators, and provide priming or activation signals for NLRP3- and caspase-11-associated inflammatory cell death [12,45]. The resulting IL-1β/IL-18 maturation and GSDMD-dependent pyroptosis may further compromise epithelial integrity and reinforce immune activation. Nevertheless, the strength of evidence differs among individual mechanisms, and the available data provide stronger support for the involvement of TLR4/NF-κB signaling and pyroptosis in specific experimental models than for a single linear signaling cascade connecting all of these pathways. Future studies should therefore combine time-resolved experiments, pathway-specific inhibitors or genetic models, microbiota manipulation, and simultaneous assessment of epithelial permeability and immune-cell responses to establish the causal hierarchy of cold-induced intestinal immune dysfunction.

### 2.4. Gut Microbiota: Cold Stress-Induced Dysbiosis and Host–Microbiota Interactions

The intestinal microbiota is an important biological component of the intestinal barrier and contributes to nutrient metabolism, epithelial integrity, immune regulation, and host energy homeostasis. Under physiological conditions, intestinal microorganisms interact continuously with epithelial and immune cells through microbial metabolites, structural components, and host signaling pathways [48,49]. Environmental temperature can influence this ecosystem indirectly through changes in host metabolism, gastrointestinal motility, nutrient availability, intestinal immune status, and the intestinal luminal environment [48]. Therefore, cold-associated alterations in the gut microbiota should be interpreted within the broader context of host–microbiota interactions rather than as an isolated microbial response.

Experimental studies have demonstrated that acute cold-related stress can rapidly alter the intestinal microbiota. In male C57BL/6 mice subjected to acute CWIR stress, intestinal mucosal injury and increased permeability were accompanied by a significant reduction in gut microbial diversity and an increase in aerobic and Gram-negative bacteria. Importantly, the abundance of these bacterial populations was associated with the severity of intestinal injury [10]. These findings indicate that acute cold-related stress can perturb microbial community structure in parallel with intestinal injury. However, because CWIR combines cold exposure with restraint and water immersion, the relative contribution of low temperature itself to the observed microbiota changes requires careful consideration.

More direct evidence has been obtained from chronic environmental cold exposure. In mice exposed to 4 °C for 3 h/day for 3 weeks, chronic cold stress significantly altered gut microbial diversity and composition and was accompanied by intestinal inflammation and pyroptosis. The abundance of several bacterial genera, including Lactobacillus, Romboutsia, Corynebacterium, and Turicibacter, was altered, and these microbial changes were associated with inflammatory and pyroptosis-related responses in intestinal tissues. Notably, the same study reported alterations in TLR4/MyD88/NF-κB and inflammasome-related signaling together with changes in microbial diversity [12]. These observations support an association between cold-induced dysbiosis and intestinal inflammatory injury; however, the available evidence does not establish that specific bacterial taxa are the primary drivers of intestinal inflammation or epithelial dysfunction.

Cold exposure may also alter the functional metabolic environment of the gut microbiota. In a mouse model in which cold water was administered intragastrically for 14 consecutive days, cold stress increased intestinal permeability and altered both microbial community structure and metabolite profiles. The microbiota showed reduced diversity and ecological network complexity, together with increased abundance of potentially pathogenic bacteria, particularly Proteobacteria [16]. These findings indicate that cold-associated dysbiosis may involve both taxonomic restructuring and changes in microbial metabolic output.

SCFAs, including acetate, propionate, and butyrate, represent important links between microbial metabolism and host physiology because they can serve as substrates for host cells and regulate epithelial, immune, and metabolic processes [48]. Experimental evidence indicates that cold exposure can alter intestinal SCFA production in a time-dependent manner. In mice exposed to 7 °C for 3 h/day for 10 days, cold exposure during the active phase increased cecal pH and reduced total SCFA and acetate concentrations, while intestinal peristalsis was also suppressed. The effects varied according to the timing of exposure, indicating that circadian phase may modify the intestinal response to cold [22]. These observations further demonstrate that cold exposure may influence microbial function without necessarily producing identical changes in microbial taxonomic composition.

The interaction between cold exposure and the gut microbiota is not unidirectional. Cold exposure increases metabolic demand and activates adaptive thermogenesis, which alters host substrate utilization and lipid metabolism. Conversely, microbial metabolites can influence host energy homeostasis and thermogenic responses [48,50]. In mice, cold exposure stimulated hepatic conversion of cholesterol to bile acids, increased fecal bile acid excretion, altered the gut microbiota, and promoted adaptive thermogenesis. Importantly, genetic or pharmacological manipulation of bile acid synthesis and biliary excretion altered both gut microbial composition and thermogenic responses [51]. These findings provide direct experimental evidence for a functional connection among host lipid metabolism, bile acid metabolism, the gut microbiota, and cold-induced thermogenesis.

Evidence from microbiota transplantation studies further supports a potential role of intestinal microorganisms in cold adaptation. Fecal microbiota from cold-tolerant Mashen pigs or cold-sensitive Duroc–Landrace–Yorkshire pigs was transplanted into mice before exposure to 4 °C for 21 days. Microbiota transplantation altered microbial composition, intestinal morphology, inflammatory responses, and SCFA concentrations in recipient mice. In particular, mice receiving microbiota from Mashen pigs exhibited greater stability of body temperature and changes in thermogenesis-associated markers, including UCP1 and PPARγ coactivator-1α (PGC-1α) [52]. These findings suggest that donor-associated microbial communities can modify aspects of the host response to cold. Nevertheless, the study did not identify a single bacterial species or metabolite responsible for the phenotype, and the mechanisms linking specific microbial taxa to cold tolerance remain unresolved.

Microbial metabolism may contribute to temperature adaptation through pathways beyond SCFAs and bile acids. In the oriental fruit fly Bactrocera dorsalis, depletion of gut microbiota markedly reduced host survival under 10 °C stress, whereas recolonization with Klebsiella michiganensis BD177 restored cold resistance and increased host arginine- and proline-related metabolites. Ribonucleic acid (RNA) interference and metabolite supplementation further supported a functional contribution of the arginine/proline metabolic pathway to low-temperature resistance [53]. Although this study provides strong causal evidence for a microbiota–metabolism–cold-resistance axis, its findings were obtained in an ectothermic insect and should therefore be considered as cross-species mechanistic evidence rather than direct evidence for mammalian intestinal cold adaptation.

The gut microbiota may also interact with the enteric neuroendocrine system during cold stress. In chickens exposed to cold stress during early life, 16S rRNA sequencing and shallow shotgun metagenomic analysis revealed persistent taxonomic and functional divergence of the cecal microbiome after the cold stimulus had ceased. Cold-exposed chickens also exhibited increased concentrations of serotonin, norepinephrine, and other monoamine neurochemicals in cecal tissue and luminal contents, and several neurochemical concentrations were associated with specific microbial taxa [54]. These findings suggest that cold-induced enteric neurochemical responses may participate in host–microbiota communication. However, the observed associations do not establish whether neurochemical changes drive microbiota remodeling or arise partly as a consequence of microbial alterations. 

Cold-induced microbial changes may subsequently influence intestinal barrier and immune function through microbial metabolites and microbial-associated molecular patterns. Changes in microbial composition and metabolic activity can affect the production of SCFAs, bile acids, amino-acid metabolites, and other signaling molecules capable of influencing epithelial integrity, mucus production, tight-junction organization, and immune-cell activity [48,49]. In chronic cold-exposed mice, changes in bacterial taxa were associated with TLR4/MyD88/NF-κB and inflammasome-related pathways, as well as markers of epithelial barrier disruption and inflammatory cell death [12]. These observations are consistent with a microbiota–barrier–immune feedback loop in which cold exposure perturbs the microbial ecosystem, microbial products or metabolites influence host responses, and barrier dysfunction subsequently alters the intestinal ecological environment.

Importantly, microbial changes observed after cold exposure should not automatically be interpreted as pathological dysbiosis. Some changes may represent adaptive remodeling associated with altered energy requirements, gastrointestinal motility, substrate availability, circadian timing, or host thermoregulation [22,50,51]. The magnitude and direction of microbial responses may also vary according to exposure temperature, duration, timing, host species, age, diet, housing conditions, and baseline microbiota. For example, intermittent cold exposure at different circadian phases produced distinct effects on intestinal SCFA concentrations [22], while early-life cold stress in chickens produced microbiome changes that persisted after the original exposure had ended [54]. These findings argue against defining a universal “cold-stress microbiota” and instead support a context-dependent model of microbial remodeling.

Taken together, current evidence supports a bidirectional relationship between cold exposure and the gut microbiota. Cold exposure can reshape microbial community structure and metabolic output through changes in host metabolism, intestinal physiology, immune status, and neuroendocrine signaling, while microbiota-derived metabolites and microbial products may influence epithelial integrity, intestinal immunity, and systemic thermogenic adaptation [51,52,53,54]. However, the causal hierarchy remains incompletely resolved, particularly in mammals. Most studies still rely heavily on 16S rRNA sequencing and correlation-based analyses, which are insufficient to determine whether specific microbial taxa directly drive intestinal injury or primarily respond to changes in the host environment. Future studies should therefore integrate longitudinal microbiome profiling with shotgun metagenomics, metatranscriptomics, microbial metabolomics, gnotobiotic or fecal transplantation approaches, and targeted manipulation of candidate microbial pathways. Such approaches will be essential for distinguishing pathogenic dysbiosis from adaptive microbial remodeling and for identifying microbiota-derived targets for prevention or intervention in cold-induced intestinal dysfunction. A concise summary of the major intestinal alterations and representative biomarkers associated with cold exposure is provided in Table 1.

## 3. Animal Models for Investigating Cold Exposure-Induced Intestinal Dysfunction

**Figure 3:**
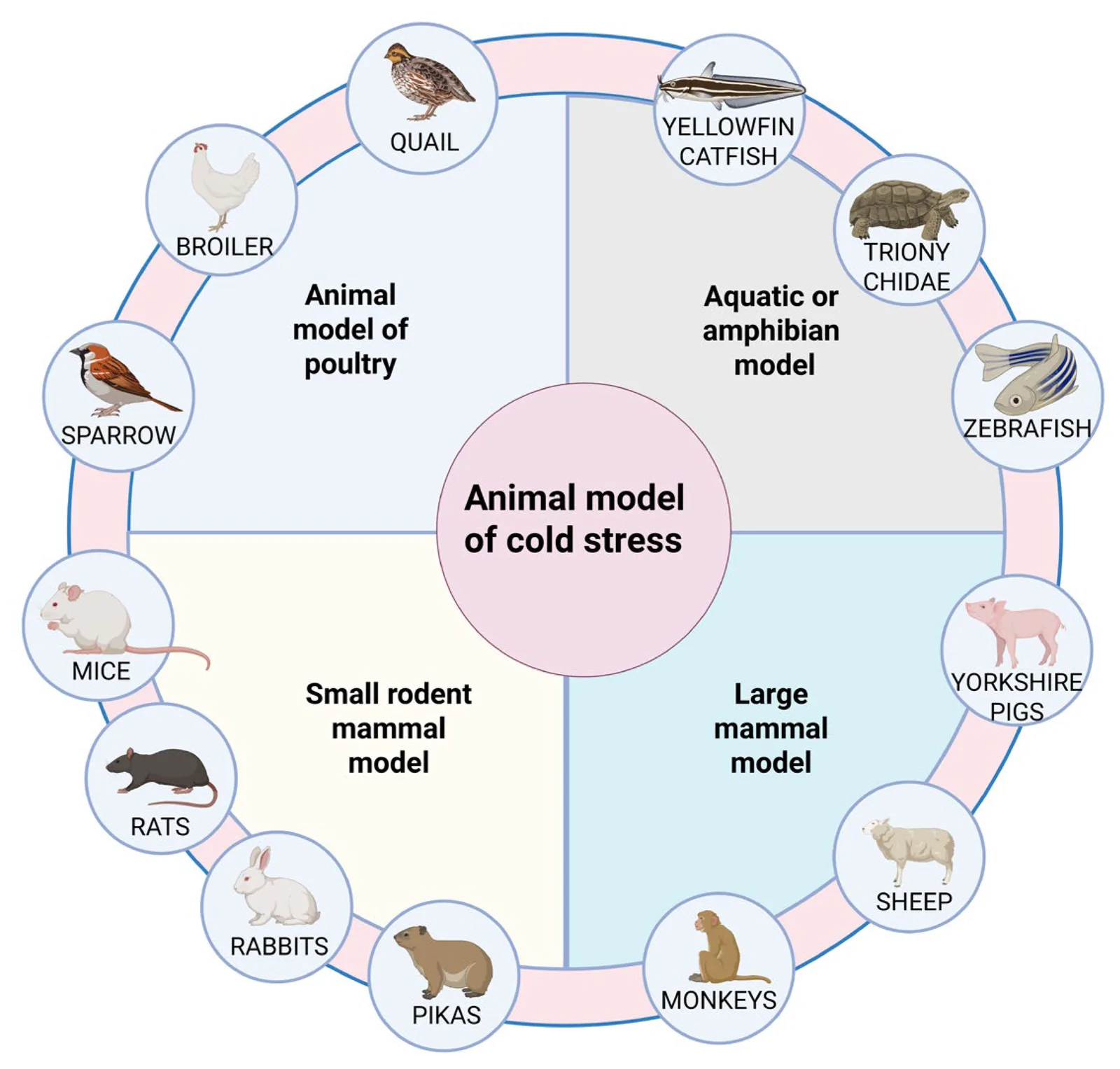
Representative animal models used to investigate cold exposure-induced intestinal dysfunction. Created in BioRender. https://app.biorender.com/illustrations/690f23d44f3bdaedbb7ad50d, accessed on 13 September 2026.

Animal models provide essential experimental platforms for investigating the physiological, metabolic, immunological, and intestinal consequences of low-temperature exposure under controlled conditions. Compared with human studies, animal experiments allow controlled manipulation of ambient temperature, exposure duration, exposure frequency, diet, and genetic background, while enabling direct collection of intestinal tissues and luminal contents. However, the biological response to cold exposure differs substantially among species because of differences in thermoregulatory capacity, body size, metabolic rate, intestinal physiology, and thermal adaptation. Endothermic mammals and birds maintain relatively stable core body temperatures through coordinated behavioral, autonomic, metabolic, and neuroendocrine responses, whereas fish are ectothermic and therefore exhibit more direct temperature-dependent changes in metabolism and physiology. Accordingly, no single animal model can reproduce the complete spectrum of intestinal responses to environmental cold. The major animal-model categories and representative species used in cold-exposure research are illustrated in Figure 3.

Rodent models are particularly useful for investigating molecular mechanisms, epithelial injury, inflammation, and host–microbiota interactions, whereas large mammals provide greater physiological and nutritional relevance to livestock production. Avian models are especially relevant to intensive poultry production because young broilers are highly sensitive to environmental temperature fluctuations, while fish provide a distinct experimental system for studying temperature-dependent metabolism, intestinal microbial adaptation, and low-temperature tolerance in ectothermic organisms. The major characteristics and applications of these models are summarized below.

### 3.1. Small Mammalian Models

#### 3.1.1. Mice

Mice are among the most frequently used mammalian models for studying cold-induced intestinal responses and systemic cold adaptation because of their well-characterized genetics, relatively low experimental cost, availability of genetically modified strains, and established methods for evaluating intestinal morphology, permeability, inflammation, oxidative stress, apoptosis, gut microbiota, and thermogenic responses.

Both acute and chronic cold-related models have been established in mice. Zhang et al. developed an acute CWIR model and demonstrated that this combined stress exposure caused severe intestinal mucosal injury, increased intestinal permeability, elevated intestinal TNF-α expression, and reduced gut microbial diversity [10]. Importantly, the study also detected thinning, shrinkage, and disorganization of intestinal villi, indicating rapid disruption of intestinal morphology. Because CWIR simultaneously incorporates cold exposure, water immersion, and restraint, however, this model should be considered a combined physical and psychological stress model rather than a pure environmental cold-exposure model [10].

More controlled environmental models have used temperature-controlled chambers. Guo et al. exposed male C57BL/6 mice to 4 °C for 3 h/day for 10 days and reported colonic structural damage, disruption of tight junctions, inflammatory activation, epithelial apoptosis, oxidative stress, and endoplasmic-reticulum stress [11]. These findings demonstrate that repeated environmental cold exposure alone can impair intestinal barrier homeostasis. A subsequent chronic cold-exposure study used 4 °C for 3 h/day for 3 weeks and reported altered gut microbial diversity, intestinal inflammation, and activation of pyroptosis-associated pathways [12].

Beyond intestinal injury, mouse models have also been extensively used to investigate cold-induced thermogenesis and systemic metabolic adaptation. Acute cold exposure activates sympathetic–adrenergic signaling and stimulates BAT, in which mitochondrial UCP1 mediates non-shivering thermogenesis and contributes to the maintenance of core body temperature [2,60]. Experimental studies have demonstrated that cold exposure increases energy expenditure and thermogenic activity in mice, with BAT activation accompanied by induction of thermogenesis-related genes and proteins, including UCP1 and PGC-1α, as well as remodeling of white adipose tissue toward a more thermogenic phenotype [61,62]. Genetic studies further support the central role of UCP1 in adaptive non-shivering thermogenesis during cold exposure [60]. More recently, mouse studies have identified additional regulators of cold-induced thermogenesis. For example, Activating transcription factor 4 (ATF4) expression in thermogenic adipocytes was shown to contribute to cold-induced thermogenesis, thermoregulation, and cold-induced browning of white adipose tissue, with effects that were dependent on nutritional status and only partly mediated through Fibroblast growth factor 21 (FGF21) [38]. Importantly, the metabolic adaptation induced by cold is not restricted to adipose tissue. In mice, cold exposure stimulates hepatic conversion of cholesterol to bile acids, which alters the gut microbiota and contributes to adaptive thermogenesis, providing a mechanistic connection between systemic metabolism, microbial ecology, and cold adaptation. These findings indicate that mouse models provide a valuable complementary platform for understanding not only cold-associated intestinal pathology but also the systemic thermogenic and metabolic adaptations that may influence intestinal physiology.

The mouse model is particularly valuable for mechanistic validation because genetic manipulation can be incorporated directly into the experimental design. For example, IL-6 knockout mice subjected to acute CWIR showed reduced intestinal epithelial injury, improved goblet-cell and permeability changes, and reduced epithelial apoptosis compared with wild-type mice, providing direct evidence for a functional role of IL-6 in acute cold-related intestinal injury [31]. Similarly, silent information regulator 2 (SIRT2)-deficient mice exposed to 4 °C for 3 h/day for 3 weeks showed aggravated colonic injury and inflammatory activation, together with increased p65 acetylation and NF-κB activation, supporting the use of genetically modified mice for dissecting specific molecular pathways [63].

The strong thermogenic capacity of mice represents both an important advantage for mechanistic studies and a limitation for translational interpretation. Mice have a relatively high surface-area-to-volume ratio and rely heavily on non-shivering thermogenesis, particularly BAT-mediated thermogenesis, to maintain core body temperature. Consequently, responses observed at temperatures such as 4 °C cannot be directly extrapolated to adult humans or livestock. Moreover, conventional laboratory housing temperatures are generally below the murine thermoneutral range and therefore impose a degree of chronic thermal challenge that increases energy expenditure and BAT activity. Experimental studies have shown that housing mice at approximately 20–22 °C increases cold-induced thermogenesis compared with thermoneutral conditions, whereas thermoneutral housing markedly reduces the requirement for adaptive thermogenesis [64,65]. Accordingly, ambient temperature should be interpreted relative to the species-specific thermoneutral range, and cold-exposure studies in mice should ideally consider core body temperature, energy expenditure, food intake, body weight, metabolic status, and acclimation history.

#### 3.1.2. Rats

Rats represent another established small-mammalian model, particularly for studying gastrointestinal physiology, epithelial proliferation, digestive function, and cold adaptation. Early work demonstrated that cold-restraint stress could induce gastrointestinal lesions in rats, establishing the sensitivity of the digestive tract to combined environmental and psychological stress.

More direct evidence was provided by Kaushik and Kaur, who subjected male Wistar rats to chronic cold stress for three weeks. Chronic cold exposure markedly reduced proliferation of crypt-base epithelial cells, altered deoxyribonucleic acid (DNA) distribution along the crypt–villus axis, increased luminal nitrite and protein concentrations, and increased intestinal myeloperoxidase activity and mast-cell numbers [55]. These observations indicate that chronic cold exposure affects both epithelial renewal and intestinal inflammatory status.

Cold exposure can also remodel the intestinal brush-border membrane and digestive enzyme profile. In chronically cold-stressed rats, intestinal brush-border membrane composition was substantially altered, while alkaline phosphatase, lactase, and leucine aminopeptidase activities increased and sucrase activity decreased. The magnitude and anatomical distribution of these changes differed among enzymes and along the crypt–villus axis [56]. These findings make the rat model particularly informative for studying the interaction between cold stress, epithelial turnover, membrane structure, and digestive function.

Rats can also be used to model physiological cold adaptation. In a chronic intermittent cold model, male Sprague–Dawley rats were exposed to 4 °C for 6 h/day for 14 consecutive days. The decline in rectal temperature after cold exposure progressively decreased during repeated exposure and became negligible toward the end of the protocol, providing evidence of physiological cold adaptation [66].

Overall, mouse and rat models are complementary. Mice are especially advantageous for genetic manipulation and pathway-specific mechanistic studies, whereas rats provide valuable models for gastrointestinal physiology, epithelial proliferation, digestive enzymes, and physiological cold adaptation.

### 3.2. Large Mammalian Models

Large mammals are particularly useful for evaluating the effects of prolonged cold exposure on whole-animal energy allocation, intestinal development, nutrient utilization, immune function, and microbiota under conditions more closely related to livestock production.

#### 3.2.1. Pigs

Pigs are among the most informative large-mammalian models of cold-induced intestinal dysfunction because of their relatively high nutritional and physiological relevance and because different breeds exhibit markedly different capacities for cold adaptation.

A particularly useful experimental design compares cold-adapted Min pigs with relatively cold-sensitive Yorkshire pigs. Teng et al. established chronic cold-exposure models in both breeds using 7 °C, with animals maintained at 17 °C in the control condition, and demonstrated clear differences between cold-adapted and non-cold-adapted phenotypes [40]. In Yorkshire pigs, chronic cold exposure was associated with impaired intestinal amino-acid transport and alterations in intestinal digestive function, whereas Min pigs showed greater physiological stability. These findings support the value of breed comparisons for investigating genetic or phenotypic differences in cold tolerance.

Another pig study exposed Yorkshire pigs to 8 ± 3 °C for 22 days and demonstrated morphological lesions in the jejunal and ileal mucosa, decreased jejunal Occludin expression, and alterations in intestinal immune homeostasis. Dietary glucose supplementation partially alleviated these intestinal abnormalities, further demonstrating the value of the pig model for evaluating nutritional interventions under chronic cold exposure [14].

Subsequent work comparing Min and Yorkshire pigs further demonstrated that chronic cold exposure altered glucose and lipid metabolism, gut microbiota, and colonic mucosal immunity, with more pronounced adverse effects in non-cold-adapted Yorkshire pigs [15]. The study also showed that cold-induced glucose overconsumption was associated with enhanced lipolysis and fatty-acid oxidation and with changes in the gut microbiota and mucosal immune environment.

Pig models therefore offer an important advantage over conventional laboratory rodents by allowing direct comparison between cold-adapted and non-cold-adapted genetic backgrounds. They are particularly suitable for investigating the interaction between cold exposure, energy metabolism, intestinal barrier function, microbiota, and nutritional status.

A further advantage of pigs is their value for translational nutritional research. Dietary glucose supplementation has been shown to improve intestinal development and immune homeostasis in cold-exposed Yorkshire pigs [14], supporting the use of pigs to evaluate feed additives and dietary strategies for maintaining intestinal function under environmental cold.

The main limitations of pig models are their higher cost, smaller feasible sample sizes, greater housing requirements, and strong dependence on breed, age, body weight, diet, and husbandry conditions. Nevertheless, their physiological and nutritional relevance makes pigs particularly valuable for studies aimed at livestock applications.

#### 3.2.2. Sheep and Other Large Ruminants

Large ruminants provide a different perspective because their digestive physiology is dominated by microbial fermentation and rumen function. Early experiments in sheep demonstrated that prolonged cold exposure altered gastrointestinal digestion and nutrient partitioning. Six sheep maintained at approximately −1 to 1 °C compared with 18–21 °C for 28 days exhibited reduced apparent digestibility of dry matter and organic matter, altered ruminal turnover, and a greater relative contribution of intestinal digestion to whole-tract nutrient utilization [67].

These findings indicate that cold exposure can modify gastrointestinal nutrient processing at the whole-animal level. Thus, sheep and other ruminant models are particularly useful for studying cold-induced changes in feed utilization, ruminal fermentation, nitrogen metabolism, gastrointestinal turnover, and energy allocation. However, because rumen fermentation dominates their digestive physiology, these models are less suitable for isolating molecular mechanisms specific to the small-intestinal epithelial barrier.

Cattle models have also become increasingly relevant for studying prolonged seasonal cold exposure. Recent work in Holstein steers under long-term cold conditions demonstrated alterations in physiological stress responses, cecal microbiota, and microbial metabolic pathways [68]. In that study, cold stress was associated with changes in microbial carbohydrate and amino-acid metabolism, while dietary oregano essential oil modified cecal microbiota and host stress-related responses. Although these findings broaden the application of large-ruminant models, direct mechanistic studies of intestinal epithelial injury remain much less extensive than those in mice, pigs, and poultry.

### 3.3. Avian Models

Birds, particularly broiler chickens, are among the most widely used livestock models for environmental cold stress. Young broilers have high metabolic requirements and rapidly increasing growth rates, while thermoregulatory capacity develops progressively after hatching. Consequently, environmental temperatures below the recommended rearing range can markedly affect growth performance, intestinal integrity, immune function, and metabolic homeostasis.

A commonly used moderate cold-stress model exposes broilers to approximately 16 °C for several days. Zhou et al. exposed 10-day-old male Arbor Acres broilers to 16 ± 1 °C for 72 h, with controls maintained at 28 ± 1 °C. Cold exposure altered growth performance, increased circulating endotoxin concentrations, and modified jejunal Occludin, ZO-1, iNOS and Adenosine monophosphate-activated protein kinase (AMPK) expression [13]. This model is therefore particularly useful for investigating the interaction between cold stress, intestinal barrier remodeling, and metabolic regulation.

More severe acute models use temperatures around 12 °C. Wu et al. exposed 7-day-old pre-starter broilers to 12 ± 1 °C for 0, 4, 8, or 12 h and examined intestinal structure together with the CIRP–TLR4–IRE1 pathway. Cold exposure increased intestinal expression of IRE1, CIRP-associated signaling components, inflammatory mediators, and tight-junction-related genes, while longer exposure reduced villus height and the villus-to-crypt ratio [32]. This model is particularly useful for studying the interaction among cold-shock responses, endoplasmic-reticulum stress, innate immune signaling, and intestinal structural injury.

Avian models can also be used to investigate physiological cold adaptation. Xing et al. exposed broilers to intermittent mild cold stimulation from day 15 to day 35 by reducing the rearing temperature by approximately 3 °C below the conventional temperature for 3 or 6 h at one-day intervals. After recovery, birds were subjected to an acute cold challenge at 10 °C for 6 h. Intermittent mild cold stimulation increased intestinal barrier-related gene expression and secretory IgA and improved subsequent resistance to acute cold stress [69].

These findings illustrate a major advantage of avian models: the same species can be used to investigate both cold-induced intestinal injury and cold acclimation. In addition, because environmental cold has direct implications for poultry growth performance, feed efficiency, disease resistance, and mortality, broiler models are highly relevant to practical livestock production.

Nevertheless, avian responses should not be directly extrapolated to mammals. Age, developmental stage, stocking density, diet, rearing temperature, and breed can strongly influence the response to cold exposure. Thus, experimental protocols should clearly report the developmental stage and thermal conditions of the birds.

### 3.4. Fish Models

Fish represent a fundamentally different model system because they are ectothermic organisms whose body temperature is closely linked to the surrounding aquatic environment. Consequently, reduced water temperature directly affects metabolic rate, feeding behavior, digestion, immune function, oxidative status, and microbial ecology. Fish models are therefore particularly valuable for investigating direct temperature-dependent physiological adaptation rather than mammalian hypothermia.

#### 3.4.1. Yellow Catfish

Yellow catfish (*Pelteobagrus fulvidraco*) are sensitive to low temperature and have been used to investigate the metabolic and intestinal consequences of cold exposure. Cold stress increased the consumption of glucose and triglycerides and enhanced the utilization of cholesterol as an alternative energy source. Transcriptomic and metabolomic analyses further demonstrated changes in thermogenesis, oxidative phosphorylation, endoplasmic-reticulum protein processing, and carbohydrate, lipid, amino-acid, and nucleotide metabolism [59].

Cold exposure also altered the intestinal microbial community of yellow catfish, including reductions in microbial abundance and increases in potentially pathogenic bacteria. These findings demonstrate that low temperature can simultaneously influence systemic energy metabolism and intestinal microbial homeostasis [54]. Consequently, yellow catfish provide a useful model for studying the interaction between environmental temperature, metabolic reprogramming, and intestinal microbiota.

#### 3.4.2. Other Fish Models

Other species provide complementary models for examining acute versus gradual cooling. In *Leiocassis longirostris*, fish were exposed to 4 °C, 26 °C, or 32 °C, and cold exposure reduced intestinal microbial richness, altered community composition, and increased the abundance of potentially opportunistic bacteria such as Aeromonas and Acinetobacter. Predicted microbial functions related to energy metabolism and cellular maintenance were also altered [70].

Silver pomfret (*Pampus argenteus*) provides a more gradual cooling model that may better approximate seasonal environmental change. Recent research simulated winter cooling from 18 °C to 6 °C using a controlled recirculating aquaculture system and demonstrated changes in feeding activity, intestinal microbial composition, and microbial community assembly during cooling [71].

Recent work in common carp (*Cyprinus carpio*) further demonstrated marked restructuring of the gut microbiota under 4 °C versus 25 °C, providing additional evidence that cold exposure can alter intestinal microbial ecosystems in ectothermic animals [72]. The study also examined antibiotic-resistance genes, broadening the potential application of fish models to environmental microbiology and One Health research.

Acute staged-cooling models have also been developed in Nile tilapia. Wipassa et al. progressively reduced water temperature from 24 °C to 14 °C, with 2 °C reductions at each stage, and measured core temperature, heart rate, ventilation rate, oxygen saturation, and cellular oxidative and mitochondrial responses. Progressive cooling reduced core temperature and ventilation and altered oxidative and mitochondrial parameters [73].

The major advantage of fish models is that they permit direct investigation of temperature-dependent metabolic and microbial responses. Their principal limitation is the fundamental difference between ectothermic and endothermic physiology. A temperature of 4 °C may constitute severe thermal stress for one species but remain physiologically tolerable for another. Therefore, low-temperature exposure should always be interpreted relative to the species-specific optimal temperature range. In addition, reduced feeding activity at low temperature can substantially affect intestinal morphology and microbiota, making feeding status and dietary intake important experimental variables.

### 3.5. Comparison and Selection of Cold-Exposure Animal Models

Taken together, these animal models are complementary rather than hierarchical. Small mammals are particularly suitable for mechanistic studies because genetic manipulation, tissue sampling, permeability assays, and microbiome analyses can be readily combined. Large mammals, especially pigs, provide greater physiological and nutritional relevance and allow investigation of breed-dependent cold adaptation. Avian models are highly valuable for poultry production research and for studying the transition between cold-induced pathology and physiological acclimation. Fish models provide a unique platform for investigating direct temperature-dependent effects on metabolism and microbial ecology in ectothermic organisms.

An important methodological consideration is that absolute exposure temperature cannot be used as a universal indicator of cold-stress severity across species. For example, mouse studies commonly use temperatures around 4 °C [11,12], broiler studies use approximately 12–16 °C [13,32], and pig studies have used approximately 7–8 °C [14,40]. These temperatures cannot be directly compared because thermoneutral and optimal temperature ranges differ substantially among species. A more appropriate approach is to describe cold exposure relative to species-specific thermoneutral or optimal temperature ranges and to consider exposure duration, exposure frequency, changes in body or core temperature, and evidence of physiological adaptation.

Future experimental studies should therefore report the animal species and strain or breed, age, sex, baseline temperature, exposure temperature, exposure duration and frequency, humidity where relevant, housing conditions, feeding status, body or core temperature, and cold-acclimation history. Standardized reporting of these parameters would improve cross-study comparability and strengthen translation of findings from animal models to human health and livestock management. The main characteristics, applications, and limitations of these models are summarized in Table 2.

Statistical power and sample size represent additional considerations when interpreting cold-exposure studies, particularly those involving large-animal models. The number of animals varies considerably among studies and is often constrained by ethical, logistical, and economic considerations. Several large-animal studies have used relatively small group sizes; for example, dietary glucose supplementation in cold-stressed Yorkshire pigs was evaluated with six animals per group. Such sample sizes may be adequate for detecting relatively large treatment effects but may provide limited power to identify modest differences or treatment-by-environment interactions. Consequently, non-significant findings in small-sample studies should be interpreted cautiously because they may reflect limited statistical power rather than the absence of a biological effect. Whenever reported, sample size, experimental unit, variance estimates, effect size, and power calculations should be considered when evaluating the strength of evidence from individual studies. Future cold-exposure experiments should incorporate prospective sample-size estimation and clearly distinguish the biological unit from technical or repeated measurements.

## 4. Intervention Strategies for Cold Exposure-Induced Intestinal Dysfunction

**Figure 4:**
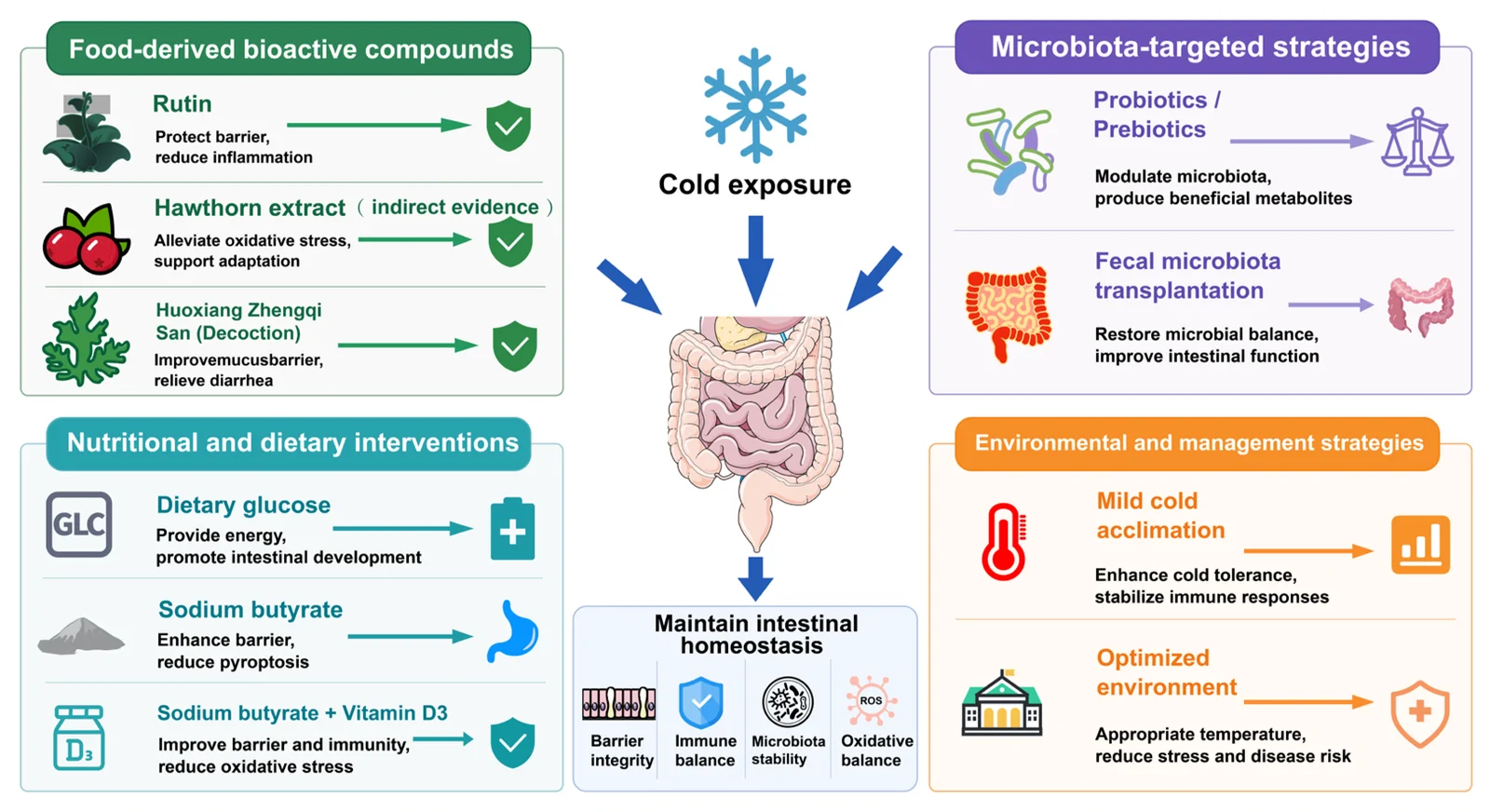
Nutritional, food-derived, and microbiota-targeted strategies for maintaining intestinal homeostasis under cold exposure.

Cold exposure-induced intestinal dysfunction is characterized by the interaction of epithelial barrier disruption, oxidative stress, inflammatory activation, altered digestive function, and gut microbial dysbiosis. Consequently, interventions directed toward a single pathological endpoint may provide incomplete protection. Current strategies include thermal restoration, food-derived bioactive compounds, nutritional and microbiota-targeted interventions, and controlled cold acclimation. However, the evidence supporting these approaches is heterogeneous. Some interventions have been directly investigated in cold-stressed intestinal models, whereas others are supported primarily by evidence from other environmental or metabolic stress conditions. Therefore, the translational relevance of each intervention should be considered according to the strength and directness of its experimental evidence. The principal nutritional, food-derived, microbiota-targeted, and environmental strategies discussed in this section are summarized in Figure 4.

### 4.1. Thermal Restoration and Rewarming

Rapid restoration of core temperature is a fundamental therapeutic strategy for clinically significant hypothermia. Depending on disease severity, passive or active external rewarming, warmed intravenous fluids, heated humidified oxygen, lavage procedures, and extracorporeal rewarming may be used to restore thermal homeostasis and cardiovascular stability. In patients with severe hypothermia and hemodynamic instability, extracorporeal rewarming can provide rapid restoration of circulation and core temperature [74].

However, rewarming is not necessarily a biologically neutral process. The transition from hypothermia to normothermia can alter tissue oxygenation, microcirculation, mitochondrial activity, reactive oxygen and nitrogen species, and apoptosis, potentially resulting in transient oxidative stress or reperfusion-like injury [74]. These responses may be relevant to intestinal tissues because restoration of circulation and oxygen availability may influence mucosal oxidative balance and epithelial recovery.

Despite its established systemic importance, direct evidence that a specific rewarming protocol prevents cold-induced intestinal injury remains limited. Most experimental and clinical studies focus on cardiovascular or neurological outcomes rather than intestinal permeability, epithelial apoptosis, or microbiota recovery. Rewarming should therefore currently be regarded as an essential supportive intervention for systemic hypothermia rather than an intestine-specific therapeutic strategy.

### 4.2. Food-Derived Bioactive Compounds and Food–Medicine Homology

Food-derived phytochemicals and food–medicine homologous substances are particularly attractive candidates for cold-induced intestinal dysfunction because they can potentially regulate multiple pathological processes simultaneously, including oxidative stress, inflammation, epithelial barrier integrity, and gut microbial homeostasis. Nevertheless, direct evidence remains limited, and compounds supported mainly by non-cold stress models should be distinguished from interventions that have been directly evaluated in cold-exposed animals.

#### 4.2.1. Rutin

Rutin is a flavonoid glycoside widely distributed in plants and food materials and has well-established antioxidant and anti-inflammatory properties. It currently represents one of the best-supported food-derived compounds for cold-induced intestinal dysfunction.

Guan et al. exposed BALB/c mice to 4 °C for 3 h/day for 21 days and administered rutin at 150 mg/kg body weight. Cold exposure impaired intestinal morphology, antioxidant status, inflammatory homeostasis, digestive enzyme activity, tight-junction-associated proteins, and gut microbial composition. Rutin significantly increased the ileal villus-to-crypt ratio, attenuated hypothermia-associated morphological injury, improved antioxidant capacity and trypsin activity, suppressed inflammatory responses, inhibited TLR4/NF-κB signaling, and restored claudin-1, occludin, and ZO-1 expression. Rutin also partially corrected the cold-induced disruption of intestinal microbiota [33]. These findings indicate that rutin may exert multi-target protection against cold-induced intestinal dysfunction.

Importantly, recent evidence has extended these findings to an avian model. In broilers exposed to cold stress, dietary rutin improved growth performance and intestinal barrier function, enhanced ileal antioxidant capacity, reduced malondialdehyde (MDA) accumulation, suppressed NF-κB-associated inflammatory responses, and modulated cecal microbiota, including reductions in potentially harmful Proteobacteria [75]. These findings strengthen the potential applicability of rutin as a dietary strategy for mitigating cold-associated intestinal dysfunction in livestock. However, further dose–response studies and independent validation in different species are required.

#### 4.2.2. Huoxiang Zhengqi San: A Food–Medicine Homologous Herbal Formula

Huoxiang Zhengqi San (HXZQS) is a traditional Chinese herbal formulation historically used for gastrointestinal disorders associated with cold–damp conditions. Unlike phytochemicals for which evidence is mainly derived from general oxidative or inflammatory models, HXZQS has been directly evaluated in experimental models of cold- and high-humidity-induced gastrointestinal dysfunction.

In a mouse model of cold and high-humidity stress, HXZQS significantly accelerated recovery from gastrointestinal dysfunction and altered intestinal digestive enzyme activity and microbiota composition. The intervention was associated with changes in intestinal lactase activity and microbial community structure, including enrichment of Lactobacillus species and restoration of microbial interaction networks [76]. More recently, Shen et al. used PacBio HiFi full-length 16S rRNA sequencing and targeted amino-acid metabolomics to investigate the mechanism of HXZQ in mice exposed to cold and humid environmental stress. HXZQ restored mucus-barrier integrity and modulated intestinal mucosal microbiota, particularly increasing *Lactobacillus reuteri* and reducing Helicobacter. Integrated microbiota–metabolite analysis further implicated glutamate and aspartate metabolism in the intervention response [77]. These findings suggest a potential microbiota–metabolism–mucus barrier axis underlying the protective effects of HXZQ.

However, these studies were conducted in models of cold–humid gastrointestinal dysfunction rather than conventional dry environmental cold exposure. Moreover, the complex multi-component composition of HXZQS makes it difficult to attribute its effects to individual compounds. Therefore, HXZQS should currently be considered a promising food–medicine homologous intervention rather than a standardized therapeutic agent for cold-induced intestinal injury.

#### 4.2.3. Hawthorn and Other Emerging Plant-Derived Interventions

Hawthorn (*Crataegus oxyacantha*) is rich in flavonoids, phenolic acids, and other bioactive compounds with antioxidant and anti-inflammatory activities. Recent experimental evidence in broilers raised under high-altitude cold stress showed that hawthorn extract increased serum superoxide dismutase (SOD) and glutathione peroxidase (GPx) activities and reduced several indices of systemic oxidative-inflammatory stress, including the heterophil-to-lymphocyte ratio [78]. These findings suggest that hawthorn may have potential for improving physiological tolerance to complex environmental stress.

However, the current evidence for hawthorn is primarily focused on systemic and cardiovascular responses rather than direct intestinal outcomes [78]. Therefore, it should not yet be presented as an established intervention for cold-induced intestinal injury. Future research should determine whether hawthorn extract can directly improve intestinal permeability, mucus production, tight-junction organization, microbiota composition, and inflammatory signaling under controlled cold-exposure conditions.

Other food-derived polyphenols, including resveratrol and quercetin, possess well-characterized antioxidant and anti-inflammatory activities and may theoretically target pathways relevant to cold-induced intestinal dysfunction. Nevertheless, for several such compounds, direct evidence in cold-exposed intestinal models remains insufficient. Evidence derived from heat stress, oxidative stress, or metabolic disease models should therefore be clearly distinguished from evidence obtained under cold exposure.

### 4.3. Dietary and Microbiota-Targeted Interventions

Cold exposure increases thermogenic energy expenditure while simultaneously affecting intestinal barrier function and microbial metabolism. Nutritional interventions may therefore protect intestinal homeostasis by improving energy availability, supporting epithelial metabolism, supplying microbial substrates, or modulating inflammatory and microbial pathways.

#### 4.3.1. Dietary Glucose and Energy Supply

Cold exposure increases energy expenditure and may alter the allocation of metabolic substrates among thermogenic tissues, skeletal muscle, and the gastrointestinal tract. Adequate dietary energy availability may therefore influence intestinal resilience.

In a pig model of chronic cold stress, dietary glucose supplementation attenuated small-intestinal mucosal injury in Yorkshire pigs. Cold exposure produced morphological lesions in jejunal and ileal mucosa, decreased jejunal Occludin expression, and increased TLR4, MyD88, NLRP3, cleaved caspase-1, mature IL-1β, and high mobility group box 1 protein (HMGB1) expression. Dietary glucose supplementation alleviated these abnormalities and reduced jejunal TLR4 and MyD88 expression [14]. These findings suggest that increasing rapidly available dietary energy can partially preserve intestinal structure and attenuate cold-associated inflammatory signaling.

The effects of glucose should nevertheless not be interpreted simply as a direct anti-inflammatory action. Increased carbohydrate availability may reduce metabolic competition among thermogenic tissues and the intestine, thereby supporting epithelial maintenance during prolonged cold exposure. Further studies are required to determine whether the intestinal protection is mediated primarily through energy availability, insulin signaling, microbial metabolism, or secondary changes in whole-body energy balance.

#### 4.3.2. Sodium Butyrate

Butyrate is a microbial short-chain fatty acid and an important energy substrate for colonocytes. It also regulates epithelial barrier function, immune responses, mitochondrial metabolism, and inflammatory signaling, making it a particularly attractive candidate for nutritional intervention.

Jiang et al. recently provided direct evidence in a cold-stressed broiler model. Broilers exposed to 10 ± 1 °C for 48 h developed intestinal histopathological damage, tight-junction alterations, oxidative stress, inflammatory activation, and pyroptosis-related changes. Sodium butyrate attenuated oxidative stress and suppressed NLRP3/Caspase-1-associated pyroptosis in cold-stressed chicken macrophages, supporting a protective role against cold-associated intestinal injury. The authors concluded that sodium butyrate supplementation mitigated cold-induced intestinal damage, potentially through suppression of oxidative stress and inflammasome-mediated pyroptosis [58].

These findings provide direct support for butyrate as a cold-stress intervention, although mechanistic attribution remains incomplete because the relative contributions of microbiota modulation, epithelial metabolism, and direct inflammasome regulation were not fully separated.

#### 4.3.3. Sodium Butyrate Combined with Vitamin D3

Recent evidence indicates that combining complementary nutritional components may provide broader protection than single supplementation. Gao et al. evaluated a diet containing 1 g/kg sodium butyrate and 2000 IU/kg vitamin D3 in Arbor Acres broilers exposed to 16 ± 1 °C for 72 h. Compared with cold-stressed birds receiving the basal diet, the combined supplementation improved average daily gain, reduced serum endotoxin and ileal IL-1β expression, increased IL-10 and Nrf2 expression, and partially stabilized the gut microbiota [79].

Histological and molecular analyses further showed improved ileal morphology and expression of genes associated with epithelial barrier function, antioxidant defense, and mucosal immunity [79]. These findings are particularly relevant to nutritional intervention because they indicate that a combination of a microbial metabolite precursor/intestinal energy substrate and an essential micronutrient may simultaneously target barrier integrity, oxidative stress, immune regulation, and microbial homeostasis.

However, because sodium butyrate and vitamin D3 were administered together, the independent contribution and possible interaction of the two components cannot be fully distinguished. Future factorial designs should therefore determine whether combined supplementation produces additive or synergistic effects.

#### 4.3.4. Prebiotics and Probiotics

Manipulation of the intestinal microbiota represents another potential approach to improving cold tolerance. Prebiotics can modify substrate availability for beneficial microorganisms, whereas probiotics directly introduce selected microbial strains. Huff et al. evaluated resistant starch prebiotics and a commercial probiotic in broiler chicks subjected to a combined cold-stress and Escherichia coli challenge. The study found that dietary resistant starch and probiotic supplementation affected body weight, feed conversion, and mortality outcomes under the combined stress model, although the responses were not uniformly beneficial across all treatment groups [80].

These findings provide preliminary evidence that microbiota-targeted nutrition may modify host responses to cold-associated stress. However, because the model incorporated an E. coli challenge in addition to cold stress, the results cannot be interpreted as direct evidence that probiotics or prebiotics protect against uncomplicated cold-induced intestinal injury. Future studies should evaluate defined microbial strains, prebiotic substrates, and postbiotics under standardized cold exposure without additional infectious challenges.

#### 4.3.5. Microbiota-Targeted Transplantation

Fecal microbiota transplantation provides a more direct approach to testing whether microbial communities contribute causally to cold adaptation. In mice receiving fecal microbiota from different pig breeds before cold exposure, transplantation altered intestinal microbial composition, microbial metabolites, SCFA concentrations, and aspects of intestinal function [52]. These findings support the possibility that microbial community structure can modify host responses to cold.

Nevertheless, the identification of a protective “cold-adapted microbiota” remains challenging because microbial communities vary substantially with host species, breed, diet, geographical location, and housing environment. Defined microbial communities and mechanistically characterized postbiotics may therefore be more readily translated than non-standardized donor-derived microbiota.

### 4.4. Cold Acclimation and Environmental Management

Unlike pharmacological or nutritional interventions, cold acclimation aims to modify the host response before severe cold exposure. Controlled, repeated mild cold stimulation may induce thermogenic, antioxidant, neuroendocrine, and immune adaptations and thereby reduce the magnitude of subsequent cold-induced injury.

In broilers, intermittent mild cold stimulation at approximately 3 °C below the conventional rearing temperature during development improved the response to a subsequent acute cold challenge. Birds receiving mild cold stimulation exhibited less intestinal inflammatory cell infiltration and structural injury and lower activation of the TLR4/MyD88/NF-κB pathway, together with improved antioxidant and immune parameters [45]. A separate study demonstrated that repeated mild cold stimulation altered intestinal cytokine and TLR expression during development, followed by recovery after the stimulation period, supporting the development of an adaptive rather than persistently inflammatory response [69].

These findings suggest that carefully controlled cold acclimation may increase resistance to subsequent severe cold exposure. However, the response is strongly dependent on temperature reduction, exposure duration, frequency, developmental stage, and recovery interval. Excessive or poorly controlled cold exposure can itself cause intestinal injury; therefore, acclimation should be viewed as a dose-dependent physiological intervention rather than a simple increase in cold exposure.

From an agricultural perspective, environmental management should complement acclimation and nutritional intervention. Maintaining appropriate thermal conditions during vulnerable developmental periods, minimizing abrupt temperature fluctuations, improving housing insulation, controlling ventilation, and ensuring adequate dietary energy supply may reduce the physiological burden imposed by cold stress. Such approaches may be particularly important for newly hatched poultry and rapidly growing livestock. The experimental evidence for these nutritional and food-derived interventions is summarized in Table 3.

### 4.5. Evidence Strength and Translational Considerations

The current intervention literature can be broadly divided into three evidence categories. Direct cold-intestine evidence is currently strongest for rutin, dietary glucose, sodium butyrate, sodium butyrate combined with vitamin D3, and selected food–medicine homologous interventions such as HXZQ/HXZQS [33,58,77,79]. Indirect or model-dependent evidence exists for probiotics, prebiotics, hawthorn, and several other plant-derived compounds [78,80]. Finally, compounds such as resveratrol and quercetin have strong biological plausibility but require substantially more direct validation in cold-exposed intestinal models before definitive conclusions can be drawn.

Another important limitation is the lack of standardized endpoints among intervention studies. Improvements in growth performance, serum oxidative markers, or inflammatory cytokines do not necessarily demonstrate restoration of intestinal function. Future intervention trials should ideally integrate histological assessment, intestinal permeability, tight-junction localization, mucus integrity, digestive function, microbial composition and function, inflammatory signaling, and whole-animal recovery. Dose–response relationships and long-term safety should also be systematically investigated.

Taken together, available evidence supports a multi-target nutritional strategy for preserving intestinal homeostasis under cold exposure. Food-derived bioactive compounds may simultaneously influence oxidative stress, inflammation, epithelial barrier integrity, digestive function, and gut microbiota, whereas dietary energy supplementation may counteract the metabolic burden associated with thermogenesis. Microbiota-targeted interventions and controlled cold acclimation provide additional preventive approaches. Nevertheless, most current evidence remains preclinical, and the field requires standardized cold-exposure protocols, mechanistically rigorous intervention studies, independent replication, and translational evaluation before these strategies can be broadly recommended.

## 5. Conclusions and Future Perspectives

Cold exposure should not be regarded as an intrinsically harmful stimulus to the intestine. Current evidence indicates that its intestinal effects are highly context dependent and may range from pathological injury to adaptive remodeling or, under selected conditions, beneficial preconditioning. Severe or prolonged cold exposure can impair epithelial integrity, alter mucus and digestive function, enhance oxidative and inflammatory responses, and disturb the gut microbiota. In contrast, controlled or intermittent mild cold exposure may promote physiological adaptation and improve tolerance to subsequent cold stress. Experimental colitis models further suggest that cold exposure may, under specific conditions, preserve epithelial integrity and promote mucosal regeneration and antimicrobial defense. Thus, the biological consequences of cold exposure depend on the interaction among exposure intensity, duration and pattern, acclimation history, host physiological status, and disease context rather than on temperature alone.

Several challenges remain before the mechanisms and translational significance of these responses can be fully established. Future studies should adopt standardized and physiologically relevant cold-exposure protocols, incorporate longitudinal assessment of injury, adaptation, and recovery, and distinguish primary intestinal effects from secondary systemic, hemodynamic, metabolic, and thermoregulatory responses. Greater emphasis should also be placed on causal validation of host–microbiota interactions, adequate statistical power, and integrated functional intestinal endpoints. Nutritional and food-derived interventions require further dose–response, safety, and translational evaluation. Overall, defining the boundary between pathological cold stress and beneficial adaptive preconditioning will be essential for understanding when cold exposure compromises intestinal homeostasis and when it may instead promote resilience.

## Figures and Tables

**Table 1:** Summary of intestinal alterations and representative biomarkers associated with cold exposure.

Biological Component	Major Alterations	Representative Biomarkers	Main Intestinal Consequences	Representative References
Physical barrier	Epithelial injury; tight-junction remodeling; altered permeability	ZO-1, Occludin, Claudins	Impaired epithelial integrity; increased permeability	[10,11,13]
Chemical barrier	Goblet-cell and mucus alterations; changes in digestive enzyme activity	MUC2, goblet cells, trypsin, lactase, sucrase	Reduced mucus protection; altered digestion	[40,55,56]
Immune barrier	Increased inflammatory responses; immune imbalance	TNF-α, IL-6, IL-1β, IL-10	Mucosal inflammation and immune dysregulation	[12,45,57]
Pyroptosis/cell death	Inflammasome activation; pyroptotic responses	NLRP3, ASC, Caspase-1, GSDMD	Epithelial injury; inflammatory amplification	[58]
Gut microbiota	Altered microbial diversity, composition, and metabolites	α-diversity, SCFAs, bile acids, microbial taxa	Dysbiosis; altered host–microbiota interactions	[16,22,59]

**Table 2:** Characteristics and applications of animal models used to investigate cold exposure-induced intestinal dysfunction.

Species/Model	Representative Cold-Exposure Protocol	Major Intestinal Outcomes	Main Mechanistic Characteristics	Main Advantages	Major Limitations	Key References
Mouse	Acute/chronic cold; CWIR or environmental cold	Barrier injury, Increase in penetration rate, inflammation, dysbiosis	TLR4/NF-κB, MAPK, NLRP3/pyroptosis, SIRT1/Nrf2	Genetic manipulation; mechanistic validation	Small-animal physiology differs from humans	[10,11,12]
Rat	Chronic cold	epithelial proliferation regression; altered BBM enzymes	Oxidative/inflammatory responses	Good physiological model	Limited genetic tools	[55,56]
Pig	Chronic low-temperature exposure	Mucosal injury, TJ changes, altered metabolism	TLR4/MyD88, microbiota–metabolism	High translational/nutritional relevance	Cost; breed differences	[14,15,41]
Sheep/Ruminants	Prolonged cold	Altered digestion and nutrient utilization	Rumen/energy metabolism	Ruminant physiology	Less molecular evidence	[67,68]
Broiler	Acute/chronic cold	Villus injury, barrier remodeling, inflammation	AMPK, TLR4/NF-κB, CIRP/IRE1	High production relevance	Age/breed dependent	[13,32,45]
Yellow catfish	Low-temperature exposure	Microbiota and energy remodeling	Metabolism–microbiota interaction	Ectothermic adaptation	Limited mammalian translation	[59]
Other fish	Gradual/acute cooling	Microbiota restructuring, metabolic adaptation	Temperature-dependent regulation	Aquaculture relevance	Cannot directly compare temperature with endotherms	[70,71,73]

**Table 3:** Experimental evidence for nutritional and food-derived interventions against cold exposure-induced intestinal dysfunction.

Intervention	Experimental Model	Cold Exposure	Intervention Dose/Protocol	Duration; Sample Size	Main Outcomes	Evidence Level	References
Rutin	BALB/c mice	4 °C, 3 h/day	150 mg/kg BW, oral administration	21 days; *n* = 10/group	Improved ileal villus-to-crypt ratio, antioxidant capacity, trypsin activity, tight-junction proteins, inflammatory responses, and gut microbiota	Direct intestinal evidence	[33]
Rutin	Arbor Acres broilers	12 °C from day 14 to 28	500 or 1000 mg/kg diet	28 days; *n* = 6 replicates/group	Improved growth performance, intestinal barrier function, ileal antioxidant capacity, inflammatory responses, and cecal microbiota	Direct intestinal evidence + growth outcomes	[75]
Huoxiang Zhengqi San (HXZQS)	KM mice with cold–high-humidity-induced gastrointestinal dysfunction	4 ± 0.5 °C, 90 ± 2% RH, 4 h/day	Decoction; 0.35 mL/mouse per administration, twice daily	7-day modeling + 3-day treatment; *n* = 15/treatment group	Improved gastrointestinal dysfunction and altered intestinal digestive enzymes and microbiota	Direct intestinal evidence; cold–humid model	[76]
Huoxiang Zhengqi Decoction (HXZQ)	Mice with cold–humid environmental stress	4 ± 0.5 °C, 90 ± 2% RH, 4 h/day	5.67 g/(kg·day), twice daily	7-day modeling + 3-day treatment; *n* = 11/treatment group	Improved mucus-barrier integrity and intestinal mucosal microbiota; increased *Lactobacillus reuteri*	Direct intestinal evidence; cold–humid model	[77]
Dietary glucose	Yorkshire pigs	8 ± 3 °C	10% glucose in diet	22 days; *n* = 6/group	Improved jejunal and ileal morphology and reduced cold-associated TLR4/MyD88 signaling and inflammatory/pyroptosis-related responses	Direct intestinal evidence	[14]
Sodium butyrate	Danzhou chickens and HD11 chicken macrophages	10 ± 1 °C, 48 h	1 mM in HD11 cells	48 h; *n* = 20/group for chickens	Reduced oxidative stress and NLRP3/Caspase-1-associated pyroptosis; intestinal protection was supported by the animal model, whereas intervention-specific mechanistic evidence was obtained in vitro	Direct intestinal + mechanistic in vitro evidence	[58]
Sodium butyrate + Vitamin D3	Arbor Acres broilers	16 ± 1 °C, 72 h	1 g/kg sodium butyrate + 2000 IU/kg vitamin D3	72 h; *n* = 8/replicate, 6 replicates/group	Improved intestinal morphology and selected barrier, immune, antioxidant, endotoxin, and microbiota-related outcomes; individual effects of the two components could not be separated	Direct intestinal evidence; combined intervention	[79]
Probiotics/Prebiotics	Broiler chicks subjected to cold stress + *E. coli* challenge	Cold-stress challenge model	Resistant starch at 15% of diet during week 1; probiotic administered in feed and water	3 weeks; 430 chicks across three studies	Modified body weight, feed conversion, mortality, and production outcomes; responses were inconsistent across treatments	Indirect/model-dependent evidence	[80]
Hawthorn extract	Ross 308 broilers at high altitude	High-altitude cold stress	0.2 or 0.4 mL/L in drinking water	42 days; 225 birds total	Improved serum SOD and GPx and reduced systemic oxidative-inflammatory and cardiovascular stress; intestinal outcomes were not directly evaluated	Systemic/indirect evidence	[78]
Resveratrol	*Oreochromis mossambicus* (tilapia)	15 °C	Resveratrol-supplemented diet	3 days of cold challenge; *n* not reported in the reviewed manuscript	Enhanced hepatic metabolic/antioxidant responses and maintained physiological cold tolerance; no direct intestinal outcome was demonstrated	Systemic/non-intestinal evidence	[81]
Fecal microbiota transplantation	Pig-to-mouse transplantation	4 °C	Donor-derived fecal microbiota, twice daily	3-day transplantation followed by 21-day cold exposure; *n* not reported in the reviewed manuscript	Altered intestinal microbial composition, microbial metabolites, SCFAs, and aspects of intestinal function	Mechanistic microbiota evidence	[52]
Mild cold acclimation	Female Arbor Acres broilers	10 °C, 24 h acute challenge	3 °C below normal rearing temperature, 5 h every other day, days 15–35	21 d; total *n* = 384 (*n* = 96/group); intestinal samples *n* = 6/group	Reduced intestinal inflammation and microvilli disruption; improved antioxidant status and sIgA; attenuated TLR4/MyD88/NF-κB signaling and inflammatory responses	Direct intestinal; adaptive preconditioning	[45]

## Data Availability

No new data were created or analyzed in this study. Data sharing is not applicable to this article.

## Abbreviations

The following abbreviations are used in this manuscript:

AIF	Apoptosis-inducing factor
AMPK	Adenosine monophosphate-activated protein kinase
ASC	Apoptosis-associated speck-like protein containing a CARD
ATF4	Activating transcription factor 4
BAT	Brown adipose tissue
CIRP	Cold-inducible RNA-binding protein
COX-2	Cyclooxygenase-2
CWIR	Cold water-immersion restraint
DNA	Deoxyribonucleic acid
ERK	Extracellular signal-regulated kinase
FGF21	Fibroblast growth factor 21
GPx	Glutathione peroxidase
GRP78	Glucose-regulated protein 78
GSDMD	Gasdermin D
HMGB1	High-mobility group box 1 protein
HXZQS	Huoxiang Zhengqi San
IgA	Immunoglobulin A
IFN-γ	Interferon-gamma
IKK	IκB kinase
IκB	Inhibitor of κB
IL-1β	Interleukin-1β
IL-2	Interleukin-2
IL-6	Interleukin-6
IL-8	Interleukin-8
IL-12	Interleukin-12
IL-17	Interleukin-17
IL-18	Interleukin-18
iNOS	Inducible nitric oxide synthase
IRAK	Interleukin-1 receptor-associated kinase
IRE1	Inositol-requiring enzyme 1
JNK	c-Jun N-terminal kinase
LPS	Lipopolysaccharide
MAPK	Mitogen-activated protein kinase
MDA	Malondialdehyde
MUC2	Mucin 2
MyD88	Myeloid differentiation primary response 88
NF-κB	Nuclear factor kappa B
NLRP3	NOD-like receptor family pyrin domain-containing 3
Nrf2	Nuclear factor erythroid 2-related factor 2
PGC-1α	Peroxisome proliferator-activated receptor gamma coactivator 1-alpha
PPARγ	Peroxisome proliferator-activated receptor gamma
RNA	Ribonucleic acid
SCFAs	Short-chain fatty acids
SIRT1	Sirtuin 1
SIRT2	Sirtuin 2
SOD	Superoxide dismutase
Th1	T helper 1
Th2	T helper 2
Th17	T helper 17
TLR4	Toll-like receptor 4
TNF-α	Tumor necrosis factor-alpha
TRAF	TNF receptor-associated factor
UCP1	Uncoupling protein 1
ZO-1	Zonula occludens-1
